# A New Highly Conserved Antibiotic Sensing/Resistance Pathway in Firmicutes Involves an ABC Transporter Interplaying with a Signal Transduction System

**DOI:** 10.1371/journal.pone.0015951

**Published:** 2011-01-19

**Authors:** Stéphanie Coumes-Florens, Céline Brochier-Armanet, Annick Guiseppi, François Denizot, Maryline Foglino

**Affiliations:** 1 Laboratoire de Chimie Bactérienne (UPR9043), Institut de Microbiologie de la Méditerranée (IFR88), CNRS, Marseille, France; 2 Université de la Méditerranée, Marseille, France; 3 Université de Provence, Marseille, France; Auburn University, United States of America

## Abstract

Signal transduction systems and ABC transporters often contribute jointly to adaptive bacterial responses to environmental changes. In *Bacillus subtilis*, three such pairs are involved in responses to antibiotics: BceRSAB, YvcPQRS and YxdJKLM. They are characterized by a histidine kinase belonging to the intramembrane sensing kinase family and by a translocator possessing an unusually large extracytoplasmic loop. It was established here using a phylogenomic approach that systems of this kind are specific but widespread in Firmicutes, where they originated. The present phylogenetic analyses brought to light a highly dynamic evolutionary history involving numerous horizontal gene transfers, duplications and lost events, leading to a great variety of Bce-like repertories in members of this bacterial phylum. Based on these phylogenetic analyses, it was proposed to subdivide the Bce-like modules into six well-defined subfamilies. Functional studies were performed on members of subfamily IV comprising BceRSAB from *B. subtilis*, the expression of which was found to require the signal transduction system as well as the ABC transporter itself. The present results suggest, for the members of this subfamily, the occurrence of interactions between one component of each partner, the kinase and the corresponding translocator. At functional and/or structural levels, bacitracin dependent expression of *bceAB* and bacitracin resistance processes require the presence of the BceB translocator loop. Some other members of subfamily IV were also found to participate in bacitracin resistance processes. Taken together our study suggests that this regulatory mechanism might constitute an important common antibiotic resistance mechanism in Firmicutes. [Supplemental material is available online at http://www.genome.org.]

## Introduction

Survival of microorganisms in their natural habitat depends on their ability to cope with fast environmental changes by controlling parameters such as ionic strength and osmotic pressure, making use of the nutriments available and resisting any toxic compounds present in the environment. Microorganisms have developed sophisticated signal transduction systems whereby extracellular stimuli are detected by membrane-integrated sensors [1]. The signals generated by these sensors are then usually transmitted across the cytoplasmic membrane *via* a phosphorylation cascade [2]. In the simplest systems of this kind, which are known as two-component systems, a two-step phosphorus transfer process is effected by a histidine protein kinase (HK) and a response regulator protein (RR). Kinases were recently classified in three major groups based on their structural properties [1]. The first and largest group consists of extracellular sensing kinases with a large extracytoplasmic detection domain. The second group is composed of kinases in which 2 to 20 transmembrane segments are connected by very short linkers. These kinases are able to detect membrane or membrane-associated stimuli and have therefore been called intra-membrane sensing kinases. The third group contains kinases with a cytoplasmic sensor domain. Once a stimulus has been sensed, the kinase autophosphorylates a conserved histidine residue present in its transmitter domain. The phosphoryl group is subsequently transferred to a conserved aspartate residue in the regulator receiver domain, which controls the expression of target genes [3]. There exist other, more complex regulatory systems involving the activation of a four-step phosphorylation cascade via extra receiver and transmitter domains [4].

Transport proteins also play an important role in microorganisms' adaptation to their environment by carrying the substrates detected across the cell cytoplasmic membrane. The role of these proteins is not restricted to transport and in some cases, they may also transmit information. There exist increasing evidences that signal transduction systems can be associated with transporters acting as co-sensors [5], [6]. Among the transporters, those of the ABC type are widespread, since they are present in all living organisms and they constitute one of the largest protein families [7]. These transporters are membrane proteins that hydrolyse ATP and thus energize the translocation of various solutes (such as ions, sugars, amino acids, vitamins, peptides, polysaccharides, hormones, lipids and xenobiotics, etc.) across the cell membrane [7], [8], [9]. ABC transporters are usually composed of two nucleotide binding domains (NBDs), which bind and hydrolyse ATP, and two membrane spanning domains (MSDs), which have also been called permease or translocators, containing multiple transmembrane segments [10]. In bacteria, ABC transporters are usually encoded by genes that are part of the same or neighbouring operons [11].

**Figure 1:**
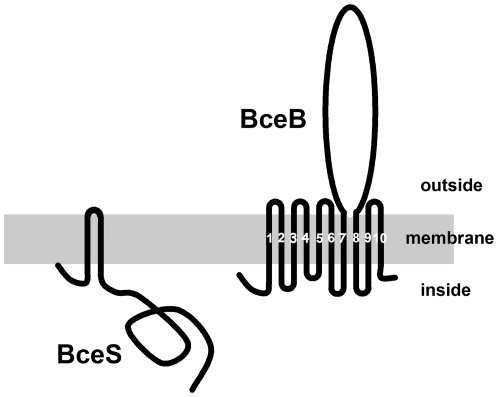
Predicted membrane topologies of histidine kinase and ABC transporter translocators in *Bacillus subtilis* Bce-like modules. The three pairs of *B. subtilis* signal transduction system/ABC transporters: Bce, Yxd and Yvc systems. Transmembrane segments (TM) are numbered from 1 to 10 in the translocator. The extracytoplasmic loop between the two TMs of the kinase varied in length from 3 to 11 residues, whereas TM7 and TM8 of the translocators are separated by 197 to 213 residues.

The *Bacillus subtilis* BceRSAB proteins involved in bacitracin resistance constitute one of the most fully studied systems in which an ABC transporter (BceAB) is combined with a signal transduction (BceRS) system [6]. It has been established in recent experimental studies that bacitracin response of course requires the BceRS signal transmission system but also a functional BceAB ABC transporter [12] and therefore involves an original and complex regulatory process. The *B. subtilis* BceAB belongs to ABC transporter family 9, all the members of which are thought to be involved in antibiotic resistance [13]. The genome of *B. subtilis* encodes two additional systems homologous to BceRSAB, namely YvcRSPQ and YxdLMJK, the expression of which is induced by enduracidin and cathelicidin LL-37, respectively. Interestingly, these three systems show several common features. First, each system is encoded by neighbouring genes on the chromosome. Secondly, the BceS, YvcQ and YxdK kinases are composed of two nearly contiguous transmembrane segments (TMS) separated by a short extra-cytoplasmic linker (consisting of less than 11 amino-acids, Figure 1). These kinases therefore belong to the intra-membrane sensing histidine kinase family [1]. Lastly, the BceB, YvcS and YxdM MSD components harbour exactly 10 TMS with an unusually large extracytoplasmic loop (from 197 to 213 residues) located between helix number 7 and helix number 8 (Figure 1). It was recently suggested that this loop might play a crucial role in the response of *B. subtilis* BceRSAB to bacitracin [14]. The functional association between an ABC transporter and a two-component system showing the different features described above will be referred to from now on as the Bce-like module.

In this study, phylogenetic analysis was performed on the four components of the Bce-like modules [13] present in the complete genomes available. These modules are widespread in Firmicutes (i.e. low G+C Gram-positive bacteria) and apart from one exception, they are restricted to this bacterial phylum, where they probably originated. The presence of multiple Bce-like copies observed in many Firmicutes suggests that these modules might contribute importantly to antibiotic resistance in this bacterial phylum. We also established that horizontal gene transfer and duplication/loss events have played an important role in the evolutionary history of Bce-like modules, leading to the acquisition of new antibiotic resistance mechanisms. Based on the present analyses, a phylogenetic classification of Bce-like modules is proposed, which could serve as a reference for functional analyses.

To further investigate the role of the ABC transporter and of the large extracytoplasmic MSD loop in the signal transduction process, Bce-like module translocators belonging to the same subfamily as the *B. subtilis* BceB were investigated. Using *B. subtilis* as a recipient, several members of this group were found to be involved in bacitracin resistance mechanisms. It was also observed that the presence of the large BceB loop is essential not only to the bacitracin dependent expression of *bceAB* but also to the resistance of the bacteria to this antibiotic.

## Results

### Taxonomic distribution and genetic organisation of the four components of the Bce-like modules

A search was performed for homologues of each of the four Bce-like module components in the 778 completely sequenced genomes available at the NCBI in February 2009 and a phylogenetic analysis was then conducted using maximum likelihood methods. This search yielded 999, 579, 1000 and 314 homologues of BceR (RR), BceS (HK), BceA (NBD) and BceB (MSD), respectively. The BceR and the BceS homologues were mainly found to exist in Bacteria, whereas BceA homologues were also present in Archaea, whereas the taxonomic distribution of BceB homologues was restricted to Firmicutes and to one Spirochaete (i.e. *Treponema denticola* ATCC 35405) (Table S1).

Among the 579 BceS homologues collected, 212 showed the same architectural features as *B. subtilis* BceS, i.e. exactly two TMS separated by a loop consisting of 12 amino acids or less. This number did not increase significantly when the threshold loop length was set at 14, since 214 homologues were detected in this case. The 212 HK showing the same architectural features as BceS will be referred to hereafter as BceS-like. Interestingly, with only a few exceptions, BceS-like sequences formed a monophyletic group in the maximum likelihood BceS tree (Figure S1). Apart from this cluster, however, most of the BceS homologues showed different features (i.e. either more or less than two TMs segments and/or longer loops, Figure S1). This suggests that all the BceS homologues in which there are two TMs connected by a short linker have a single evolutionary origin. The non canonical architectural features observed in a few BceS-like sequences such as those found to occur in *Lysinibacillus sphaericus* (YP_00169681) and *Bacillus halodurans* (NP_241142) represented therefore secondary modifications, whereas the presence of a few BceS-like sequences outside this group probably resulted from sporadic convergences (Figure S1). Similar findings and conclusions can be reached on the 293 MSD sequences which, like *B. subtilis* BceB, were found to harbour exactly ten TMS and to have a long extracytoplasmic loop (i.e. more than 170 amino acids) located between TMS numbers 7 and 8 (Figure S2). Interestingly, BceS-like and BceB-like families showed similar taxonomic patterns of distribution: apart from one exception, they were both restricted to and specific to Firmicutes (Figures S1 and S2).

The phylogenies of the two remaining components (RR and NBD) harboured a monophyletic cluster, which was quite similar in terms of the taxonomic distribution to those obtained with BceS-like and BceB-like homologues (data not shown). The sequences recorded in these clusters will be referred hereafter as BceR-like and BceA-like.

**Figure 2:**
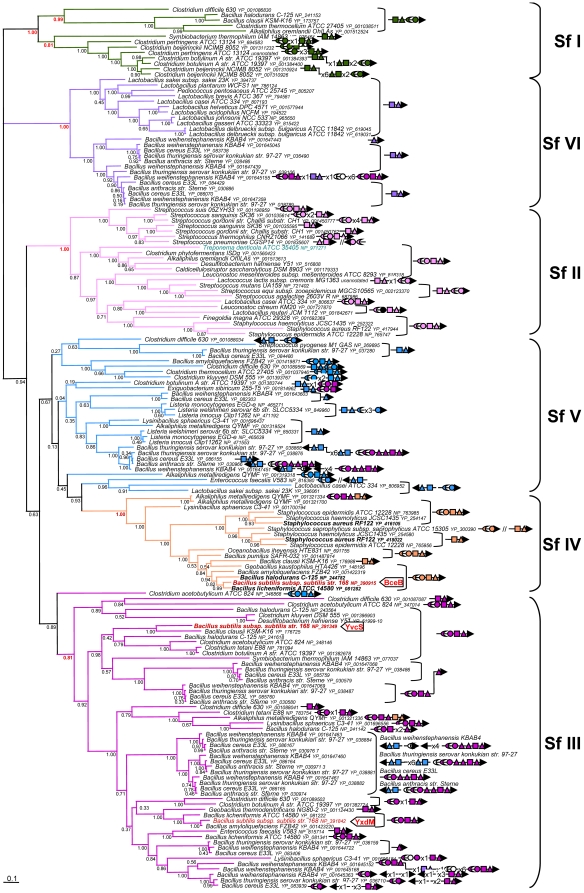
Phylogeny of the BceB-like proteins (MSD components). Bayesian tree showing the relationships in a subsample of 164 BceB-like sequences. The accession number of each sequence is provided. Numbers at nodes are posterior probabilities. The scale bar indicates the average number of substitutions per site. Based on the phylogeny of each component, six subfamilies (numbered from I to VI) were defined; they are indicated here by brackets and colours. Symbols represent Bce-like components encoded by genes located in the neighbourhood of *bceB*-like homologs: triangles correspond to MSD, crescents to regulators, circles to kinases, squares to NBD, and numbers indicate the number of non *bce*-like genes. The arrow indicates the direction of transcription. Color of the symbols indicates to which family they belong and corresponds to the color of the tree branchs (green I; light pink II; dark pink III; orange IV; blue V, purple VI, whereas black is used to designate unclassified homologs and white symbols indicate non homologous regulators, kinases, NBDs or MSDs). BceB, YvcS and YxdM from *B. subtilis* homologs are indicated in red and by a thick arrow. The unique non Firmicutes BceB-like homologue from *T. denticola* is shown in light blue. The length of the alignment used to construct the tree was 251 residues. BceB-like proteins used for recombinant strain construction were indicated in bold characters.

**Figure 3:**
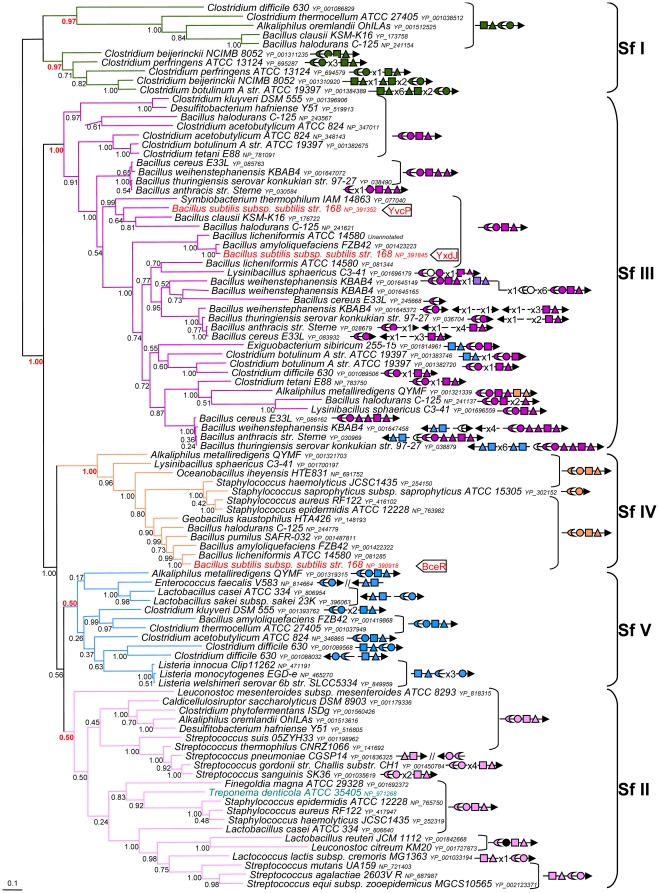
Phylogeny of the BceR-like proteins (regulators). Bayesian tree showing the relationships in a subsample of 96 BceR sequences. The accession number of each sequence is provided. Numbers at nodes are posterior probabilities. The scale bar indicates the average number of substitutions per site. For details about colours and symbols, see the legend to Figure 2. The length of the alignment used to construct the tree was 187 residues.

The fact that the taxonomic distribution of the Bce-like component is restricted to Firmicutes strongly suggests that they originated in this phylum. To pursue this point further, a Bayesian phylogenetic analysis was performed on each Bce-like component. Although the four resulting trees were not completely resolved, they showed very similar topologies (Figures 2–3 and Figures S3–S4). This indicates that the four components underwent the same evolutionary history after their emergence in Firmicutes.

This hypothesis was supported by our survey of the genomic sequences, which showed that apart a few exceptions, the genes encoding a *bce*-like component formed a cluster containing at least one copy of each component (Table S1). The fact that their physical proximity in the genomes was highly conserved might account for the co-evolution of the genes encoding Bce components, which was highlighted by the results of the present phylogenetic analyses. These findings also suggest that ancestors of these genes already formed a single cluster corresponding to a functional module.

Interestingly, although most clusters contain only one gene copy of each *bce*-like element, some of them contain several genes coding for MSD and/or NBD components (Table S1). Most of these additional copies (e.g. the three MSD sequences of *Bacillus weihenstephanensis* KBAB4 (YP_001647460, YP_001647461 and YP_001647463) resulted from recent gene duplication events, Figure 2). This suggests either that some signal transduction systems might control the expression of several ABC transporter coding genes or that ABC transporter might be formed by heteroduplex rather than simple homodimer (NBD-MSD)_2_. Another atypical situation was previously encountered with *bceA*- and *bceB*-like genes such as SalX (NP_269896) and SalY (NP_269895) from *Steptococcus pyogenes* which have been found to be involved in salivaricin resistance [15]. These genes are clustered together with genes encoding for RR and HK that are not homologous to *B. subtilis* BceS and BceR. This indicates that a secondary association of genes coding for ABC transporters was formed with regulatory/transduction coding genes from other systems.

### Toward a classification of Bce-like modules

Based on the phylogenetic analysis of the components of Bce-like modules, these modules were subdivided into six subfamilies. Subfamily I was observed in trees based on MSD, RR and NBD (branches in green, Posterior Probability (PP)  = 1.00, 1.00 and 0.74 respectively, Figures 2–3 and Figure S4) and mainly included sequences from Clostridiales. In the kinase based tree, this subfamily consisted of two parts (Figure S3), possibly due to a rather weak overall resolution of this phylogeny. Subfamily II was mainly found in Lactobacillales and Clostridiales, but also in some Spirochaetes sequences (branches in light pink, PP = 1.00 (MSD), 0.50 (RR), 1.00 (HK) and 1.00 (NBD), Figures 2–3 and Figures S3–S4). The emergence of *Treponema denticola* ATCC 35405 Bce-like sequences in subfamily II indicated that this spirochaete had acquired the four genes from a single Firmicutes donor via a single horizontal gene transfer. This subfamily was also found to include the BceRSAB module (components NP_721401 to 721404) and the YP_001835606, YP_001835607 ABC transporter, which are known to participate in bacitracin resistance in *Streptococcus mutans* [16], [17] and *S. pneumoniae* [18], [19], respectively. Subfamily III was mainly present in Clostridiales and Bacillales (branches in dark pink, PP = 0.81 (MSD), 1.00 (RR), 1.00 (HK), 0.81 (NBD), Figures 2–3 and Figures S3–S4). However, the corresponding group in the NBD tree also included a few sequences belonging to the fifth subfamily (see below) (Figure S4). This subfamily included sequences from the Yxd and Yvc modules of *B. subtilis* which are activated by LL-37 and enduracidin, respectively. Subfamily IV contains the components of the *B. subtilis* Bce module, as well as sequences coding for the VraFG ABC transporter (YP_416104, YP_416105) coupled to the GraRS two-component system (YP_416102, YP_416103) from *Staphylococcus aureus,* which are involved in vancomycin resistance [20]. This subfamily was found to occur mainly in Bacillales (branches in orange, PP = 1.00 in the fourth trees, Figures 2–3 and Figures S3–S4). Subfamily V had a widespread pattern of occurrence in Firmicutes and included sequences from Clostridiales, Bacillales and Lactobacillales. This was the least strongly supported family, which was only detected and weakly supported in mainly the BceR tree (branches in blue, PP = 0.50, Figure 3), however in the other trees, the corresponding sequences often occurred close together, which suggests that they might be related. The BceA-like and BceB-like trees contained the AnrAB ABC transporter (NP_465638, NP_465639) which has been reported to be involved in *Listeria monocytogenes* bacitracin resistance [21]. They also included MSD and NBD components of the ABC transporter which are encoded in *Streptococcus pyogenes* genomes by *salX* and *salY* (NP_269895 and NP_269896, Figures 2 and S4. See also Table 1). Contrary to the other Bce-like modules on which functional data are available, the *S. pyogenes* transporter is not involved in antibiotic resistance, but in antibiotic biosynthesis. More specifically, the corresponding genes were found in a cluster involved in salivaricin biosynthesis which, in addition to *salX* and *salY*, contained the *salA* salivaricin structural gene, the *salB* and *salT* enzyme modification genes, and *salR* and *salK*, encoding a signal transduction system which is not homologous to BceRS. Lastly, subfamily VI was mainly found to occur in Bacillales and Lactobacillales. Unlike the other subfamilies, it contains only BceB- and BceA-like components (purple branches, PP = 1.00 in both trees, Figure 2 and Figure S3). This means that no BceR- or BceS-like sequences belong to this family.

**Table 1:** Bacitracin resistance of various *B. subtilis* strains.

− IPTG		+ IPTG		Ratio +/−
Strains	mean ± SD	Strains	mean ± SD	
BSGY005	281±61	pDG*bceR*	316±28	1.1
Δ*bceAB*	1±0.4	Δ*bceAB,* pDG*bceR*	1.3±0.3	nc
*BceABBs*_ri_ (*B. subtilis* NP_390916/NP_390915)	98±22	*bceABBs*_ri_, pDG*bceR*	142±19	1.4
*YtsCDBl*_ri_ (*B. licheniformis* YP_081283/YP_081282)	90±22	*ytsCDBl*_ri_, pDG*bceR*	133±7	1.5
*BceABBh*_ri_ (*B. halodurans* NP_244781/NP_244782)	13±0.8	*bceABBh*_ri_, pDG*bceR*	30±4	2.3
*VraDESa*_ri_ (*S. aureus RF122* YP_418021/YP_418022)	1.4±0.3	*vraDESa*_ri_, pDG*bceR*	2±1.1	nc
*VraFGSa*_ri_ (*S. aureus RF122* YP_416104/YP_416105)	1.2±0.5	*vraFGSa*_ri_, pDG*bceR*	1.1±0.1	nc
*bceBBs*Δloop (*B. subtilis* NP_390915 Δloop)	1.9±0.1	*bceBBs*Δloop, pDG*bceR*	3.2±1.5	nc
*ytsDBl*Δloop (*B. licheniformis* YP_081282 Δloop)	1±1	*ytsDBl*Δloop, pDG*bceR*	1.5±0.9	nc
*BceB*loopBceB*Bs*_ri_ (*B. subtilis* NP_390915 with *B. subtilis* NP_390915 loop)	110±11	*bceB*loopBceB*Bs*_ri_, pDG*bceR*	140±23	1.3
*BceB*loopYtsD*Bl*_ri_ (*B. subtilis* NP_390915 with *B. licheniformis* YP_081282 loop)	2.8±1.4	*bceB*loopYtsD*Bl*_ri_, pDG*bceR*	2.4±1.1	nc
*BceB*loopBceB*Bh*_ri_ (*B. subtilis* NP_390915 with *B. halodurans* NP_244782 loop)	2.5±0.5	*bceB*loopBceB*Bh*_ri_, pDG*bceR*	2.4±0.9	nc
*BceB*loopYvcs*Bs*_ri_ (*B. subtilis* NP_390915 with *B. subtilis* NP_391349 loop)	1.4±0.2	*bceB*loopYvcs*Bs*_ri_, pDG*bceR*	1.7±1.2	nc

The names of the proteins introduced into *B. subtilis* to completely or partly replace the *B. subtilis* BceAB transporter and the species to which they belong are indicated between brackets on the left of the table. All MSD proteins are members of sub-family IV except NP_391349, which belongs to sub-family III. Strains (_ri_ means that the corresponding ABC transporter was reconstituted/introduced into the appropriate *B. subtilis* background, see Table S2) were grown in medium alone (−IPTG) or in medium containing 1 mM IPTG (+ IPTG) to induce *bceR* expression and then tested to determine their bacitracin resistance in 96-well microtiter trays [22]. At the end of the incubation period, OD at 600 nm was monitored using a TECAN microtiter tray reader. IC50 is defined as bacitracin concentration (µg.ml^−1^) giving 50% growth inhibition. Results are expressed as mean IC50 values obtained in at least 3 different experiments ± standard deviations. The ratio (IC50 with IPTG (+)/IC50 without IPTG (−)) is given in the case of IC50 values >5 (nc: not calculated).

The similarities in the topology of these four trees indicate that the components have undergone similar evolutionary histories and thus, that they have co-evolved. It is worth noting that in each subfamily, the phylogeny of the sequences did not follow the phylogeny of the corresponding species. For example, *Staphylococcus* sequences belonging to subfamily II were more closely related to Lactobacillales and Clostridiales sequences than to sequences of other Bacillales (Figures 2–3, and Figures S3–S6). In addition, the taxonomic distribution of these subfamilies showed that closely related species may harbour very different gene repertories. For instance, *Geobacillus thermodenitrificans* contained only components from subfamily III, whereas its close relatives *Geobacillus kaustophilius* and *Anoxybacillus flavithermus* harboured only components from subfamily IV or no components at all, respectively (Figure S5b). The great disparity between organisms and gene phylogeny suggest that the genes coding for these components have been extensively transferred during the evolution of Firmicutes. It is also difficult to trace the evolutionary history of Bce-like modules because of gene duplication events such as those involving MSD components from *Bacillus anthracis* belonging to subfamily III (Figure 2) and because of gene losses: our detailed survey of complete genomes brought to light a number of degenerated sequences, which were annotated as pseudogenes (some of them were tested experimentally) (Table S1). This suggests that although the acquisition of genes coding for components of the Bce-like system occurred relatively frequently as the result of horizontal gene transfer and/or gene duplication events, these genes were also frequently lost, which would explain the great variability observed among the bce-like genes. Accordingly, the evolutionary history of these genes must have been highly complex and it cannot be completely explained. In particular, it is impossible to say from which lineage of Firmicutes the ancestral Bce-like module emerged. However, the wide pattern of distribution shown by this module among Firmicutes suggests that these systems play an important functional role in this group of bacteria.

This clarification of the evolutionary relationships between Bce-like modules should provide a good starting point for functional studies as indicated below.

### Determining the role of the ABC transporter: the case of subfamily IV

#### 1) At the bacitracin resistance level

In subfamily IV, several transporters have been either found or thought to confer bacitracin resistance to the bacterium in which they are expressed. This was found to be the case with BceAB from *B. subtilis* [22]–[24] and might also the case with its closest homologues, YtsCD (YP_081283 and YP_081282) from *B. licheniformis* because it has been established that *ytsCD* genes are up-regulated in the presence of bacitracin [25]. It has also been suggested that VraDE (YP_418021 and YP-418022) may participate in bacitracin resistance in *S. aureus* [26]. Contrasting with the specificity of subfamily IV members toward bacitracin, VraFG (YP_416104 and YP_416105) from *S. aureus* also respond to vancomycin [20]. BceAB (NP_244781 and NP_244782) from *B. halodurans*, the functional role of which has not yet been elucidated, are very closely related to *B. subtilis* BceAB. To further investigate how they contribute to bacitracin resistance, the corresponding genes were introduced into *B. subtilis* at the *bceAB* locus under the control of the *bceAB* promoter (*YtsCDBl*_ri_, *BceABBh*_ri_, *VraDESa*_ri_ and *VraFGSa*_ri_ strains, Table S2). 6 His codons were added at the 5′ terminus of the transporter's NBD coding genes to facilitate the detection of the protein (see Material and Methods). Control assays were carried out using a strain in which the *B. subtilis bceAB* genes were reintroduced into *B. subtilis* using the same procedure (*BceABBs*_ri_ strain). All strains were then tested to determine their bacitracin IC50 levels (the bacitracin concentration giving a 50% growth inhibition). In comparison with the BSGY005 strain, which includes a wild type *bce* locus (IC50 = 281±61 µg.ml^−1^, Table 1), the control *bceABBs*_ri_ strain was found to be less resistant (IC50 = 98±22 µg.ml^−1^, Table 1). This difference might be due to the addition of a His6-tag at the NH2 terminus of the BceA NBD protein. As all the strains contained an NBD gene expressing a protein bearing this slight modification, their IC50 were compared with that of the *bceABBs*_ri_ strain IC50. The *ytsCDBl*_ri_ strain showed the same level of bacitracin resistance (IC50 = 90±22, Table 1) suggesting that the *B. licheniformis* YtsCD ABC transporter may have a similar function to that of the *B. subtilis* BceAB ABC transporter. A lower level of resistance was observed with the *bceABBh*_r_ strain (IC50 = 13±0.8, Table 1). Surprisingly, the *vraDESa*_ri_ strain and the *vraFGSa*_ri_ both showed no resistance to bacitracin: both strains had similar IC50 values to that of the Δ*bceAB* strain (IC50≤1.4 µg.ml^−1^, Table 1). A decrease in ABC transporter expression might explain either the partial or complete absence of complementation observed with the various strains. To test the ABC transporter efficiency without any bacitracin induction, pDG*bceR* was introduced into the various strains and BceR was overproduced by IPTG induction. These conditions were found to mimic the bacitracin signal and the *bceS* deleted strain overproducing BceR was as resistant to bacitracin as the WT strain (unpublished data). Western blot analysis with a His-tag detection system showed that under IPTG induction conditions, each reconstituted ABC transporter was synthesized in a fairly similar level to that of the wild type strain and was expressed at membrane level (data not shown). No significant effects of IPTG on the IC50 values were detected in any of the strains except for *bceABBh*_ri_, where a faint increase in the IC50 was observed (IPTG fold induction  = 2.3, Table 1), which indicates that this reconstituted ABC transporter is partially functional in *B. subtilis*. All in all, the results of these experiments suggest that a) bacitracin resistance decreases with the phylogenetic distance between BceAB and the substituted transporter (from *B. licheniformis* YtsCD to *S. aureus* VraDE), although the amounts of reconstituted ABC transporter at the cell membrane in the corresponding strains were fairly similar to those of the wild type strain; b) it was established here for the first time to our knowledge that YtsCD*Bli* and BceAB*Bha* confer bacitracin resistance on the bacteria in which they are expressed.

#### 2) At the bacitracin induction level

To test the ability of the reconstituted ABC transporter to participate in P*bceAB* promoter activation in the presence of bacitracin, a P*bceAB::lacZ* transcriptional fusion was introduced at the available *amyE* locus in all the strains (WT, Δ*bceAB*, *bceABBs*_ri_, *ytsCDBl*_ri_, *bceABBh*_ri_, *vraDESa*_ri_ and *vraFGSa*_ri_). The activity of this fusion was monitored when the strains were grown in either the presence or absence of bacitracin.

Without bacitracin, almost no β-galactosidase activity (≤0.6±0.4 unit) was detected in any of the strains (Table 2). When bacitracin (4 µg.ml^−1^) was added to the cell culture, no β-galactosidase activity (≤0.2 unit) was observed in *vraDESa*_ri_ or *vraFGSa*_ri_ strains, whereas *bceABBs*_ri_ and *ytsCDBl*_ri_ strains were perfectly able to respond even more strongly to bacitracin than in the case of the BSGY005 strain (61±15units, 38±6 units and 39±11 units, respectively). Lastly, a very faint β-galactosidase activity was observed when the *bceABBh*_ri_ strain was grown in the presence of bacitracin (2±1units, Table 2).

**Table 2:** β-Galactosidase specific activity of various *B. subtilis* strains.

	Bacitracin SD	
	0 µg.ml^−1^	4 µg.ml^−1^
Strains		
BSGY005	0.6±0.2	39±11
Δ*bceAB*	0.6±0.4	1±1
*bceABBs*_ri_	0.6±0.3	54±9
*ytsCDBl*_ri_	0.4±0.1	38±6
*bceABBh*_ri_	0.3±0.2	2±1
*vraDESa*_ri_	0.3±0.1	0.2±0.1
*vraFGSa*_ri_	0.3±0.1	0.1±0.1
*bceBBs*Δloop	0.5±0.1	0.6±0.3
*ytsDBl*Δloop	0.5±0.4	0.6±0,2
*bceB*loopBceB*Bs*_ri_	0.7±0.2	75±15
*bceB*loopYtsD*Bl*_ri_	0.7±0.1	10±4
*bceB*loopBceB*Bh*_ri_	0.4±0.5	0.6±0.2
*bceB*loopYvcs*Bs*_ri_	0.3±0.2	0.3±0.2

Strains (_ri_, see Table 1), containing a P*bceA::lacZ* transcriptional fusion at the *amyE* locus, were grown for 1 hour in LB medium with and without 4 µg.ml^-1^ of bacitracin as indicated. β-galactosidase specific activities are given as the mean values obtained in at least 3 experiments ± standard deviations.

These results show that a) the presence of the His tag at BceA NH2 terminus did not hamper the ability of the reconstituted ABC transporter to produce a sustained response to bacitracin b) the response decreased rapidly as the phylogenetic distance between the replacing transporter and *B. subtilis* BceAB increased, and only *B. licheniformis* YtsCD, the most closely related ABC transporter, was able to even partially compensate for this decrease.

### The presence of the large extra-cytoplasmic BceB loop is essential to bceAB promoter induction and bacitracin resistance

To determine the functional role of the BceB loop, a *B. subtilis bceB*Δloop strain and a *B. subtilis* strain in which the encoding BceB loop region was reintroduced into *bceB* using the procedure described in Material and Methods (*bceB*loopBceB*Bs*_ri_ strain) were compared in terms of their ability to activate the *bceA* promoter in the presence of bacitracin: the two strains tested here carried a P*bceAB::lacZ* transcriptional fusion at the *amyE* locus. As was to be expected, no significant β-galactosidase activity above the background levels (0.7±0.2 unit) was detected in any of these strains when bacitracin was omitted (Table 2). In the presence of bacitracin (4 µg.ml^−1^), almost no activity was recorded in the *bceB*Δloop (0.6±0.3 unit, Table 2), whereas a greater level of response than that recorded with the BSGY005 strain (containing a wild type *bce* locus) occurred in the *bceB*loopBceB*Bs*_ri_ strain (75±15 units, Table 2). This shows that the BceAB ABC transporter was completely restored by reintroducing the BceB loop. These results show that normal bacitracin induced *bceAB* expression requires the presence of the BceB loop.

The bacitracin IC50 value was then determined in the two strains mentioned above. The IC50 value of the *bceB*Δloop strain was similar to that of the Δ*bceAB* strain (1.9±0.1 µg.ml^−1^, Table 1). This value was more than two orders of magnitude lower than the IC50 of the BSGY005 strain (around 280 µg ml-1). This result is in good agreement with the inability of the *bceB*Δloop strain to activate the P*bceAB* promoter in the presence of bacitracin (Table 2) and thus to synthesize the BceABceBΔloop transporter. All these strains were then tested under *bceR* over-expression conditions and the *bceB*Δloop pDG*bceR* strain was found to be as sensitive to bacitracin (IC50 = 3.2±1.5 µg.ml^−1^) as the Δ*bceAB* pDG*bceR* strain (Table 1). However, the *bceB*loopBceB*Bs*_ri_ pDG*bceR* strain and the *bceABBs*_ri_ pDG*bceR* strain used as a control showed practically the same significant increase in bacitracin resistance (IC50 values around 140 µg.ml^−1^, Table 1), which indicates that bacitracin resistance in *B. subtilis* requires the presence of the BceB loop.

### The Δloop BceAB ABC transporter is expressed at membrane level

**Figure 4:**
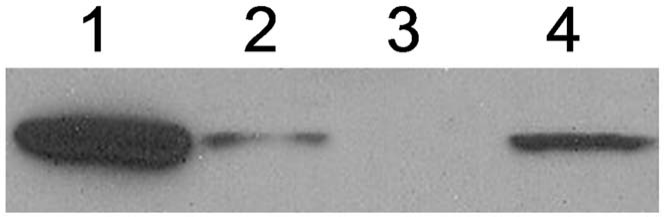
BceA cellular localisation followed by Western blot analysis. The *bceB*Δloop pDG*bceR* strain was grown in medium containing IPTG (1 mM). Cells were disrupted and subjected to sub-cellular fractionation. Lane 1, crude lysate; lane 2, supernatant resulting from low speed centrifugation; lane 3, supernatant resulting from high speed centrifugation; lane 4, pellet resulting from high speed centrifugation (membrane fraction). Western blots were probed with a rabbit anti-BceA antibody.

The lack of bacitracin resistance observed in the *bceB*Δloop, pDG*bceR* strain grown in medium containing IPTG might be attributable to a defective BceAB ABC transporter insertion into the bacterial membrane. Western blots obtained using an anti-BceA polyclonal antibody clearly showed the presence of the BceA protein in the IPTG induced *bceB*Δloop pDG*bceR* strain, both in the crude extract (Figure 4, lane 1) and in the cell lysate obtained after eliminating the cell debris (Figure 4, lane 2). Upon subjecting the cell lysate to sub-cellular fractionation and testing the sub-fractions by performing Western blot experiments, no BceA protein was recovered in the supernatant obtained after ultracentrifugation (Figure 4, lane 3), but this protein was clearly detected in the pellet corresponding to the membrane fraction (Figure 4, lane 4). The nucleotide binding protein of an ABC transporter interacts tightly and specifically with its MSD membrane partner [27]. Therefore, the fact that BceA was detected in the membrane preparation suggested that the entire BceAB ABC transporter was correctly located in the cytoplasmic membrane despite the absence of the BceB loop.

This strongly suggests that correct localisation of the transporter in the cellular membrane does not require the BceB loop, although the presence of this loop is required to obtain a completely functional ABC transporter for both bacitracin induction and resistance processes in *B. subtilis*.

### The loops of the most homologous BceB are poorly conserved

The question then arose as to whether other loops in subfamily IV ABC transporters involved in responses to bacitracin might play a similar functional role to that of the large extra-cytoplasmic *B. subtilis* BceB loop. When the *B. licheniformis* YtsCD transporter deleted from the MSD large loop was expressed in *B. subtilis,* both the responses and resistance to bacitracin were abolished in the bacterium, which shows that as in the case of *B. subtilis* BceAB, the loop was required to obtain a fully functional ABC transporter (Table 1 and Table 2). We therefore examined the seven closest relatives of BceB, which formed a well defined branch in the MSD tree (i.e. proteins from *B. subtilis, B. licheniformis*, *B. amyloliquefaciens*, *B. halodurans*, *B. pumilus*, *G. kaustophilus*, *B. clausii* and *O. iheyensis*, Figure 2 *)* with a view to characterizing their large extra-cytoplasmic loops. The corresponding MSD sequences were aligned using the Muscle software program. The resulting alignment showed that contrary to expectations, the large extracytoplasmic loop (corresponding to O4) was less highly conserved than other regions of these proteins (i.e. other loops or TMS) (Figure S6). To obtain a closer picture, we computed the similarity between BceB from *B. subtilis* and that from the seven other proteins in each region of the proteins. The large extracytoplasmic loop O4 was systematically found to be the least highly conserved element, giving a mean identity of 0.38±0.1, whereas the values obtained in the other regions ranged between 0.52±0.13 (O2) and 0.88±0.07 (TM7) (Figure S7). This finding was at odds with the possible functional or structural importance of the loop.

Did the loop have to be present simply to serve as a particular linker or did it confer its substrate specificity to the ABC transporter? With a view to answering these questions, the BceB loop from *B. subtilis* was replaced by either the *B. licheniformis* YtsD loop, the *B. halodurans* BceB loop or the distantly related *B. subtilis* YvcS loop (subfamily III), using the appropriate strategy for obtaining the *bceB*loopBceB*Bs*_ri_ strain. After introducing the P*bceAB::lacZ* transcriptional fusion at the *amyE* locus, these strains were tested to determine their ability to respond to bacitracin. Except for the *bceB*loopBceB*Bs*_ri_ strain (see above), only the *B. licheniformis* YtsD loop induced partial recovery of the response, whereas both the *B. halodurans* BceB loop and the *B. subtilis* YvcS loop failed to restore the response to bacitracin (Table 2). It is worth noting that the *bceB*loopYtsD*Bl*_ri_ strain overexpressing BceR was not found to be resistant to bacitracin (Table 1) even if the chimeric ABC transporter was membrane detected by western blot experiments (data not shown). These results indicate clearly that a) the functional and/or structural importance of the MSD loop does not depend only on the presence of a protein fragment with an appropriate length b) only the most closely related loop (*B. licheniformis* YtsD loop) partly restored the functional integrity of *B. subtilis* BceB c) the entire BceB MSD loop was required to obtain a completely functional ABC transporter.

## Discussion

The results of the present comprehensive study on *B. subtilis* Bce, Yvc and Yxd module homologues in complete genomes clearly show that 1) these modules composed of a two-component signal transduction system (with an intra-membrane sensing HK) combined with an ABC transporter (with an MSD possessing an unusually large extra-cytoplasmic loop) are restricted in Firmicutes with a wide occurrence in this phylum; 2) the four components (RR, HK, NBD and MSD) composing Bce-like modules have co-evolved since they emerged in Firmicutes; 3) the Bce-like module coding genes have been frequently transferred, duplicated and/or lost during their evolution; and 4) the chromosomal proximity between their respective genes has been highly conserved during evolution.

Based on the detailed phylogenetic analyses performed here, a system of classification of the Bce-like modules into six subfamilies was proposed. Most of the functional data available on Bce-like modules so far have suggested that these modules may be involved in antibiotic resistance mechanisms (against bacitracin, vancomycin, LL-37, enduracidin, etc.). This might explain the crucial role played by horizontal gene transfer and duplication/loss events occurring during their evolutionary history which have lead to the emergence of a large repertory of Bce-like module in some Firmicutes such as pathogenic *Bacillus* or *Clostridia* (Figure S5–S6).

To further investigate the functional links existing between the various components of these modules, we studied several members of the Bce-like subfamily IV, some of whose ABC transporters proved to be involved in bacitracin resistance mechanisms (*B. subtilis* BceRSAB, *S. aureus* VraDE) or to be up-regulated in the presence of bacitracin (*B. licheniformis* YtsCD). The Δ*bceAB B. subtilis* strain was complemented with genes encoding these modules. In all the bacitracin induction and bacitracin resistance tests performed, the same pattern was consistently observed: the responses of the strains decreased rapidly with the phylogenetic distance between *B. subtilis* BceAB and the replacing ABC transporter. At the induction level, this pattern may be attributed to the need for protein interactions during the process. The ability to interact may indeed decrease when one of the two partners are replaced by a more phylogenetically distant protein. This is in line with our previous suggestion that interactions between one of the BceAB subunits and BceS may activate the HK and induce the response to bacitracin [12]. We have previously described the crucial role of UPP in the response of *B. subtilis* to bacitracin, and suggested that a UPP/bacitracin complex rather than bacitracin alone might participate in the activation of the BceRS system [12]. Undecaprenyl pyrophosphate (UPP) is the bacterial molecular target of bacitracin [28], and its sequestration by the antibiotic leads to the inhibition of peptidoglycan biosynthesis, which is lethal to the bacterium. How does the presence of bacitracin result in BceS kinase activation? As mentioned above, we have hypothesized that interactions may occur between the transporter and the kinase [12]. BceAB might therefore recognize the UPP/bacitracin complex and then make a change of conformation. In this new conformational state, BceAB might interact with and activate BceS. In this case, BceAB would be the first proteins to sense the presence of bacitracin. Might this antibiotic induction mechanism apply to all Bce-like modules? Some of the data available seem to suggest that this is the case: other modules seem to function like the *B. subtilis* Bce module, such as the Yvc module, which requires the presence of the YvcRS ABC transporter to induce a response to enduracidin in *B. subtilis* (our unpublished data). Likewise, in the Bce module from *S. mutans,* the BceAB transporter acts as a bacitracin co-sensor with the BceRS two-component [16]. Also, the *S. aureus* GraRS signal transduction system up-regulates the expression of the genes coding for the VraFG ABC transporter in the presence of vancomycin, and this process seems to require the presence of at least VraG [20].

Despite the fairly high levels of membrane expression of the various ABC transporters, their ability to protect bacteria from bacitracin decreases or is lost when they are expressed under heterologous conditions. Differences in the cellular context, such as changes in the peptidoglycan structure or the protein environment, might explain this finding. For instance, the VraDE transporter, which was previously found to participate in *S. aureus* bacitracin resistance, fails to complement BceAB in *B. subtilis*, whereas *B. licheniformis* YtsCD and *B. halodurans* BceAB, two closer homologues of BceAB, at least partly restore bacitracin resistance in *B. subtilis*. It is worth mentioning that these are the first experimental data supporting the putative contribution of these two transporters to bacitracin resistance in their respective genuine bacterial hosts. The up-regulation of *B. licheniformis ytsC* gene found to occur in the presence of bacitracin in both *B. licheniformis DSM13* and *ATCC10716* strains supports these findings [26]. At least four ABC transporters belonging to subfamily IV recognize bacitracin as a substrate: BceAB*Bs*, YtsCD*Bl*, BceAB*Bh* and VraDE*Sa* [26 and the present results). This specificity is not restricted to subfamily IV, since BceAB (NP_721401, NP_721402) in *Streptococcus mutans,* YP_001835606 and YP_ 001835607 in *Streptococcus pneumoniae,* both of which belong to subfamily II, and the subfamily V AnrAB proteins (NP_465638, NP_465639) from *Listeria monocytogenes* confer bacitracin resistance on their respective bacterial hosts [16], [17], [18], [19], [21]. This indicates that very distantly related modules can have the same specificity. In addition, members of the same subfamily can have different specificities, such as VraFG*Sa,* which belongs to subfamily IV and was found to contribute to vancomycin resistance in *S. aureus* [20]. These data indicate that the antibiotic recognized by each Bce-like module may be difficult to predict on the basis of phylogenomic data, with the exception of some very close relatives. However, the fact that the target of all the antibiotics involved in known modules is the bacterial cell envelope constitutes a common feature among all of them.

The specificity of the ABC transporter is puzzling because it raises questions about the recognition of the antibiotic by the MSD. This specificity can be either restricted, as in the case of the *B. subtilis* BceAB transporter, which specifically recognizes bacitracin, based on our previous results [24] or very broad, as in the case of the BceAB from *S. mutans,* the *S. pneumoniae* YP_001835606/YP_001835606 ABC transporter and the *L. monocytogenes* AnrAB transporter, all of which recognize several antibiotics [18], [19], [21]. Interestingly, some Firmicutes seem to have acquired multi-antibiotic resistant ABC transporter as the result of either gene duplication, horizontal gene transfer and/or recombination events leading to the extension of the Bce-like module's repertory. This seems to have already occurred in some bacteria, especially in human pathogenic bacteria such as *S. mutans* and *S. pneumoniae* (see above) or *B. cereus* and *B. anthracis* (Figures 2 and S4), which suggests that they are better equipped to resist several antibiotics.

Upon examining the sequence of the *B. subtilis* BceB's closest homologues belonging to subfamily IV, we noted that the loop is the least conserved domain. This contrasts with our finding that the presence of the loop is required not only for induction but also for antibiotic resistance processes. When the *B. subtilis* BceB loop was replaced by loops from subfamily IV ABC transporters with the same specificity, none of the heterologous ABC transporter loops was found to restore bacitracin resistance to this bacterium. Although the loop might be required for the proper folding and activity of the transporter to occur, the possibility that it might have a more specific function cannot be ruled out. Indeed, the most closely related loop (the *B. Licheniformis* YtsCD loop) partially restored the bacitracin response in the *B. subtilis* BceBΔloop strain. The occurrence of interactions between the loop and some other domain(s) in the transporter might explain the latter result and in this context, phylogenetically distantly related loops might loose their ability to take part in these interactions. Large extracytoplasmic loops have been previously found to be involved in specific functions in an ABC transporter belonging to the exporter family in the case of *E. coli,* where LolC and LolD along with the LolD NBD constitute the LolCDE ABC transporter. This transporter carries out the first step in lipoprotein transport from the inner to the outer membrane of the Gram negative bacteria [29]. It has been predicted that LolE and LolC each contain four TMS and a large extracytoplasmic loop located between TMS number 1 and 2. These loops interact directly with lipoproteins and are involved in their transfer to the LolA periplasmic molecular chaperone [30], [31]. It therefore seems possible that the BceB-like loops might also constitute important functional domains for the corresponding ABC transporter.

### Conclusions

The results obtained throughout the present study consistently indicate that Bce-like modules are involved in resistance mechanisms against antibiotics targeting the membrane. The data obtained here, along with those available in the literature, suggest that a common mechanism of action is involved. These modules include an ABC transporter possessing a translocator with a very large extracytoplasmic loop, which seems to be functionally and/or structurally required by all of them. In addition, the translocator seems to act as the primary antibiotic sensor, which transfers the information it detects to the histidine kinase. This unusual mode of functioning makes these particular Bce-like modules interesting targets for further studies. Better knowledge about this resistance mechanism and how these modules developed and spread in Firmicutes might provide interesting clues to designing more appropriate means of treating diseases due to pathogens belonging to this bacterial phylum.

## Materials and Methods

### Bacterial strains, plasmids and growth conditions

Bacterial strains and plasmids used in this study are listed in Tables S2 and S3, respectively. The sequence of each recombinant plasmid was checked by DNA sequencing (Cogenics). *B. subtilis* strains were grown in Luria broth (LB) at 37°C with aeration. The pDG148*bceR* plasmid [32] was used to induce over-expression of BceR in *B. subtilis*. IPTG was also added at a final concentration of 1 mM when necessary. Recombinant strains were grown in medium containing antibiotics at the following concentrations: chloramphenicol (5 µg.ml^−1^), tetracycline (10 µg.ml^−1^) and spectinomycin (100 µg.ml^−1^). Bacitracin and all other antibiotics were from Sigma-Aldrich.

### General Molecular Biology Techniques

Unless otherwise stated, all molecular biology procedures were carried out as described in [33]. DNA fragments were purified using the Qiaquick nucleotide removal kit (Qiagen). Cloning of DNA was performed in *E. coli* DH5α strain. PrimeSTAR (Takara Bio. Inc.) was used to perform PCR amplifications in a final volume of 50 µl under the conditions recommended by the manufacturer. Plasmid purifications were carried out using either Plasmid Midi Kit or Plasmid Mini Kit from Qiagen.

All the oligonucleotides used in this study are listed in Table S4.

### Plasmid and strain construction procedure

#### General strategy for *bceAB* deletion, ABC transporter gene introduction and loop exchange in *B. subtilis*

Three plasmids were designed and produced, each containing a *bceAB* DNA flanking region so that a DNA fragment could be either deleted or inserted at this locus by inducing a double recombination event.

1) The pbTy plasmid was obtained by cloning a three partner PCR product into the pGEM-T vector (Promega). PCR products were obtained as follows: PCR1 fragment (351 base pairs (bp)), comprising in the following order part of the *bceA* promoter, the *bceA* start codon followed by 6 histidine encoding codons, a *PmlI* restriction site (CACGTG, where CAC is the last histidine codon) and an *AcsI* restriction site (GGCGCGCC), was obtained using Pbcea1 and Pbcea2-ml primers. The 401 bp long PCR3 fragment including the end of the *bceB* gene and the beginning of the *yttA* gene was obtained using yttA1-tet and yttA2 primers. *B. subtilis* genomic DNA was used as a template in both cases. PCR2, which corresponds to a tetracycline resistance cassette, was obtained using Tet1-asc and Tet2-lin primers with the pDG1515 plasmid as a template [34]. PCR1/PCR2 and PCR2/PCR3 possess 20-bp and 24-bp long fragments, respectively, with identical sequences, so that amplification can be performed using pbceA1 and ytta2 as primers with a mixture of the three fragments at an equimolecular ratio. *PmlI*, *AscI* and *BstEII* (end of the Tet cassette) are unique restriction sites in pbTy.

The pbTy vector was used to construct the pbSy vector (see below) and to clone entire ABC transporter genes at *PmlI*/*AscI* sites in order to replace *bceAB* genes.

2) The pbSy plasmid was obtained from pbTy by replacing the tetracycline cassette by a spectinomycin cassette. The latter was obtained by performing PCR amplification using spec-asc and spec-bst primers and pDG1726 as a template [34]. After digestion with *AscI*/*BstEII*, the cassette was cloned into the *AscI*/*BstEII* pbTy digested plasmid, giving the pbSy plasmid. The latter was used to obtain a Δ*bceAB* mutant in *B. subtilis* via a double recombination event.

3) The pbTy*bceAB*Δloop plasmid was obtained from the pbTy plasmid. A DNA fragment comprising *bceA* gene and the *bceB* gene with the loop deleted was obtained as follows: using *B. subtilis* genomic DNA as template and bceABBs-pml with loop1/FseBbv or loop2/FseBbv with bceABBs-asc as pairs of primers, we obtained PCR4 and PCR5 fragments respectively. Since these fragments had identical 21-bp fragments, double partner amplification was performed with PCR4 and PCR5 as templates (in a 1/1 molar ratio) using bceABBs-pml and bceABBs-asc as primers. After a digestion step with *PmlI* and *AscI*, the PCR fragment was cloned into the *PmlI*, *AscI* digested pbTy plasmid, giving the pbTy*bceAB*Δloop plasmid. As the cloned fragment contained two restriction sites (*FseI*, *BbvCI*) replacing the loop encoding sequence, which were not present in the pbTy plasmid, a loop encoding sequence could be reintroduced. By reintroducing the BceB loop in this way, three additional amino acids were introduced on both sides of the loop (GLS and VLS) in comparison with the wild type sequence. The pbTyb*ceAB*Δloop plasmid was also used to obtain the *bceB*Δloop strain from the *bceAB* mutant via a double recombination event.

#### Cloning of ABC transporter genes into the pbTy plasmid and reintroducing the genes into *B. subtilis*

*bceAB* from *B. subtilis*, *ytsCD* from *B. licheniformis, bceAB* from *B. halodurans, vraDE* and *vraFG from S. aureus,* genes were obtained by PCR amplification using bceABBs-pml/bceABBs-asc, ytsCDBl-pml/ytsCDBl-asc, bceABBh-pml/bceABBh-asc, vraDE-PmlI/vraDE-asc and vraFG- PmlI/vraFG-asc as pairs of primers, respectively, with the corresponding genomic DNAs as templates. Each amplification product was *PmlI*/*AscI* double digested and cloned in the *PmlI*/*AscI* double digested pbTy plasmid. Each of the resulting plasmids was used to reintroduce the *B. subtilis bceAB* genes, *B. licheniformis ytsCD* genes, *B. halodurans bceAB* genes, *S. aureus* vraDE and *S. aureus* vraFG genes into the *B. subtilis bceAB* mutant, giving the *bceABBs*_ri_, *ytsCDBl*_ri,_ *bceABBh*_ri_, *vraDESa_ri_* and *vraFGSa_ri_* strains, respectively.

#### Cloning of sequences encoding MSD loops into the pbTy*bceAB*Δloop plasmid and reintroducing the corresponding ABC transporter genes into *B. subtilis*

*bceBBs*, *ytsDBl* or *bceBBl* loop sequences were obtained by performing PCR amplification using loop-BceBBs_fse*/*loop-BceBBs_bbv, loop-ytsD-Bl_fse/loop-ytsD-Bl_bbv and loop-BceBBh_fse/loop-BceBBh_bbv pairs of primers, respectively, with the corresponding genomic DNAs as templates. Each amplification product was *FseI*/*Bbv*CI double digested and cloned into the *FseI*/*Bbv*CI double digested pbTyb*ceAB*Δloop plasmid. Each of the resulting plasmids was used to reintroduce either the BceBBs loop, the YtscDBl loop or the BceBBh loop into the Δ*bceAB* strain, giving the *bceB*loopBceB*Bs*_ri_, *bceB*loopYtsD*Bl*_ri_, and *bceB*loopBceB*Bh*_ri_ strains.

Using this general strategy, all the strains except the Δ*bceAB* strain expressed a BceA protein possessing a 6-his N terminal tag making detection by His-probing proteins possible if required.

### β-galactosidase assay

The procedure used here has been previously described [12].

### Measurement of bacitracin resistance

The antibiotic concentration of giving 50% growth inhibition (IC_50_) was determined using the microtiter tray assay described previously [22].

### Cell lysate and membrane preparation

The procedure used here has been previously described [35].

### Western blot experiments

Proteins were separated by performing SDS-page and the gels were blotted onto Hybond ECL paper (Amersham Biosciences) using a semi-dry transfer apparatus (Bio-rad) in line with the manufacturer's recommendations. BceA protein was detected either with a rabbit polyclonal anti-BceA antibody and a second antibody (mouse anti-rabbit Ig coupled to horse radish peroxydase from Sigma-Aldrich) or with a His-probe Horseradish peroxydase (super signal west Hisprobe kit from Thermo Scientific). In both cases, the SuperSignal West Pico chemoluminescent substrate from Pierce was used in line with the manufacturer's recommendations.

### Bioinformatic analysis

Homologues of the four components of the *B. subtilis* Bce-modules were retrieved from the 779 complete genomes available in February 2009 at the NCBI using the BLASTp program [36]. Sequences from the Bce-module of *B. subtilis* (i.e. BceR (O34951) 231 amino acids, BceS (O35044) 334 amino acids, BceA (O34697) 253 amino acids, BceB (O34741), 646 amino acids) were used as seeds.

The sequences retrieved were aligned using the MUSCLE software program [37]. Resulting alignments were inspected visually and refined manually using the MUST software program [38]. Regions where alignment was doubtful were removed before the phylogenetic analyses were carried out using MUST.

Phylogenetic analyses were performed using the Maximum Likelihood method implemented in PHYML [39], with the Le and Gascuel model (LG model) including an estimated proportion of invariant sites and a correction by a Γ-law to account for rate among site variations (four categories of sites, an estimated alpha parameter). Refined phylogenetic analyses were performed on each Bce-like component, using a reduced taxonomic sampling method, where only one strain was conserved in each species. The Bayesian method implemented in MrBayes 3.0B4 [40] was used with a mixed model based on amino acid substitution and a Γ-law (four discrete categories plus a proportion of invariant sites) to account for among site rate variation. MrBayes was run with four chains for 1 million generations and trees were sampled every 100 generations. To construct the consensus tree, the first 1500 trees were discarded as burning.

Lastly, the TM segments of the BceS and BceB homologues were investigated using the filter_tmhmmv2.pl program (C. Brochier-Armanet., unpublished). Sequences were sorted depending on their length, the number of TMs, the position and the length of their extracytoplasmic loops. Among the 578 BceS homologs, we searched for sequences having a length ranging from 50 to 1000 amino acids, exactly two TM segments and a short loop (*i.e.* composed of 1–20 amino acids) located between the first and second TM segments. In the case of 314 BceB homologs, we examined sequences having a length ranging from 400 to 1000 amino acids, exactly ten TM segments and a long loop (*i.e.* composed of 100–400 amino acids) located between the seventh and eighth TM segments.

## Supporting Information

Figure S1 **Phylogeny of BceS homologues.** Maximum Likelihood tree showing the 579 BceS homologues retrieved from complete genomes. Sequences with the name in green correspond to kinases that harbour exactly 2 TM separated by a short linker (<12 amino acids), whereas sequences with the name in orange correspond to BceS with different characteristics (more or less TM, longer linker, etc). The great majority of these sequences are clustered together, which indicates that this characteristic was present in the ancestor and conserved during the evolution of this group. The few sequences outside the clusters correspond to sporadic convergences occurring during the evolution of this large family. The scale bar gives the average number of substitutions per site.
(PDF)

Figure S2 **Phylogeny of BceB homologues.** Maximum likelihood tree showing the 314 BceB homologues retrieved from complete genomes. Sequences with the name in green correspond to MSD that harbour exactly 10 TM, and TM7 and TM8 are separated by a long extracytoplasmic loop (>197 amino acids), whereas sequences with the name in orange correspond to BceB with different characteristics (more or less TM, longer linker, etc). The scale bar gives the average number of substitutions per site.
(PDF)

Figure S3 **Phylogeny of the BceS-like proteins (kinase components).** Bayesian tree showing the relationships in a subsample of 98 BceS sequences. The accession number of each sequence is provided. Numbers at nodes are posterior probabilities. The scale bar gives the average number of substitutions per site. For details about colours and symbols, see the legend to Figure 5. The length of the alignment used to construct the tree was 171 residues.
(PDF)

Figure S4 **Phylogeny of the BceA-like proteins (NBD components).** Bayesian tree showing the relationships in a subsample of 152 BceA sequences. The accession number of each sequence is provided. Numbers at nodes are posterior probabilities. The scale bar gives the average number of substitutions per site. For details about colours and symbols, see the legend to Figure 5. The length of the alignment used to construct the tree was 205 residues. BceA-like proteins used for recombinant strain construction were indicated in bold characters.
(PDF)

Figure S5 **Bce-like repertories.** Type and distribution of Bce-like systems in the four main Firmicutes lineages: (A) Clostridiales, (B) Bacillales, (C) Mollicutes and (D) Lactobacillales. For details about symbols and colours, see the legend of Figure 5.
(TIF)

Figure S6 **Alignment of the** ***B. subtilis*** **BceB sequence with its seven closest relatives belonging to subfamily IV.** Regions designated by I1 to I6 are intracellular segments, those annotated O1 to O5 correspond to extracellular loops (O4 is the large loop), and TM stands for transmembrane segments. In the line entitled “Clustal Consensus”, stars correspond to positions harbouring residues that are different but that have similar features. In this alignment, the region corresponding to O4 is the least highly conserved.
(TIF)

Figure S7 **Similarities between** ***B. subtilis*** **BceB regions and those of the closest BceB-like homologs.** The Y axis gives the evolutionary distances and the X axis gives the domain of interest in the BceB-like proteins. Whole: entire proteins, I: intracytoplasmic domains, TM: transmembrane segments, O: extracytoplasmic or outside domains. SD Numbering of the domains corresponds to the predicted topology of BceB-like proteins (Figure S6). In each region of the proteins, the mean distance between the *B. subtilis* BceB transporter and the seven most closely related BceB-like proteins belonging to subfamily IV (proteins from *B. licheniformis*, *B. amyloliquefaciens*, *B. halodurans*, *B. pumilus*, *G. kaustophilus*, *B. clausii* and *O. iheyensis*) is indicated. Error bars correspond to standard deviations.
(TIF)

Table S1 **Diversity and organisation of** ***bce*** **-like components in each complete genome.** Kinases are shown in yellow, regulators in red, NBDs in green, MSDs in blue to and non Bce-like proteins in grey. Light colors correspond to pseudogenes or misannotated genes that have been manually detected using tblastn with complete genomes. For each gene cluster, the name of the subfamily to which it belongs is indicated.
(XLS)

Table S2 **Bacterial strains used in this study.**
(PDF)

Table S3 **Plasmids used in this study.**
(PDF)

Table S4 **List of oligonucleotides used in this study.**
(PDF)

## References

[1] Mascher T (2006). Intramembrane-sensing histidine kinases: a new family of cell envelope stress sensors in Firmicutes bacteria.. FEMS Microbiol Lett.

[2] Parkinson JS, Kofoid EC (1992). Communication modules in bacterial signaling proteins.. Annu Rev Genet.

[3] Stock J, Surette M, Levit M, Par P (1995). Two-component signal transduction systems: structure-function, relationships and mechanism of catalysis..

[4] Stock AM, Robinson VL, Goudreau PN (2000). Two-component signal transduction.. Annu Rev Biochem.

[5] Tetsch L, Jung K (2009a). The regulatory interplay between membrane-integrated sensors and transport proteins in bacteria.. Mol Microbiol.

[6] Tetsch L, Jung K (2009b). How are signals transduced across the cytoplasmic membrane? transport proteins as transmitter of information.. Amino Acids.

[7] Ren Q, Kang KH, Paulsen IT (2004). TransportDB: a relational database of cellular membrane transport systems.. Nucleic Acids Res.

[8] Ames GF (1986). Bacterial periplasmic transport systems: structure, mechanism, and evolution.. Annu Rev Biochem.

[9] Higgins CF, Hiles ID, Salmond GP, Gill DR (1986). A family of related ATP-binding subunits coupled to many distinct biological processes in bacteria.. Nature.

[10] Higgins CF (1992). ABC transporters: from microorganisms to man.. Annu Rev Cell Biol.

[11] Tomii K, Kanehisa M (1998). A comparative analysis of ABC transporters in complete microbial genomes.. Genome Res.

[12] Bernard R, Guiseppi A, Chippaux M, Foglino M, Denizot F (2007). Resistance to bacitracin in *Bacillus subtilis*: unexpected requirement of the BceAB ABC transporter in the control of expression of its own structural genes.. J Bacteriol.

[13] Quentin Y, Fichant G, Denizot F (1999). Inventory, assembly and analysis of *Bacillus subtilis* ABC transport systems.. J Mol Biol.

[14] Rietkötter E, Hoyer D, Mascher T (2008). Bacitracin sensing in *Bacillus subtilis*.. Mol Microbiol.

[15] Upton M, Tagg JR, Wescombe P, Jenkinson HF (2001). Intra- and interspecies signaling between *Streptococcus salivarius* and *Streptococcus pyogenes* mediated by SalA and SalA1 lantibiotic peptides.. J Bacteriol.

[16] Ouyang J, Tian X, Versey J, Wishart A, Li Y (2010). The BceABRS Four-Component System Regulates Bacitracin-Induced Cell Envelope Stress Response in *Streptococcus mutans*.. http://dx.doi.org/10.1128/AAC.01802-09).

[17] Tsuda H, Yamashita Y, Shibata Y, Nakano Y, Koga T (2002). Genes involved in bacitracin resistance in *Streptococcus mutans*.. Antimicrob Agents Chemother.

[18] Becker P, Hakenbeck R, Henrich B (2009). An ABC transporter of *Streptococcus pneumoniae* involved in susceptibility to vancoresmycin and bacitracin.. Antimicrob Agents Chemother.

[19] Majchrzykiewicz JA, Kuipers OP, Bijlsma JJE (2010). Generic and specific adaptive responses of S*treptococcus pneumoniae* to challenge with three distinct antimicrobial peptides, bacitracin, ll-37, and nisin.. Antimicrob Agents Chemother.

[20] Meehl M, Herbert S, Götz F, Cheung A (2007). Interaction of the GraRS two-component system with the VraFG ABC transporter to support vancomycin-intermediate resistance in *Staphylococcus aureus*.. Antimicrob Agents Chemother.

[21] Collins B, Curtis N, Cotter PD, Hill C, Ross RP (2010). The ABC transporter Anrab contributes to the innate resistance of *Listeria monocytogenes* to nisin, bacitracin, and various beta-lactam antibiotics.. Antimicrob Agents Chemother.

[22] Ohki R, Giyanto, Tateno K, Masuyama W, Moriya S (2003). The BceRS two-component regulatory system induces expression of the bacitracin transporter, BceAB, in *Bacillus subtilis*.. Mol Microbiol.

[23] Mascher T, Margulis NG, Wang T, Ye RW, Helmann JD (2003). Cell wall stress responses in *Bacillus subtilis*: the regulatory network of the bacitracin stimulon.. Mol Microbiol.

[24] Bernard R, Joseph P, Guiseppi A, Chippaux M, Denizot F (2003). YtsCD and YwoA, two independent systems that confer bacitracin resistance to *Bacillus subtilis*.. FEMS Microbiol Lett.

[25] Wecke T, Veith B, Ehrenreich A, Mascher T (2006). Cell envelope stress response in *Bacillus licheniformis*: integrating comparative genomics, transcriptional profiling, and regulon mining to decipher a complex regulatory network.. J Bacteriol.

[26] Pietiäinen M, François P, Hyyryläinen H, Tangomo M, Sass V (2009). Transcriptome analysis of the responses of *Staphylococcus aureus* to antimicrobial peptides and characterization of the rôles of VraDE and VraSR in antimicrobial resistance.. BMC Genomics.

[27] Liu PQ, Ames GF (1998). In vitro disassembly and reassembly of an ABC transporter, the histidine permease.. Proc Natl Acad Sci U S A.

[28] Stone KJ, Strominger JL (1971). Mechanism of action of bacitracin: complexation with metal ion and C 55 -isoprenyl pyrophosphate.. Proc Natl Acad Sci U S A.

[29] Narita S, Tokuda H (2006). An ABC transporter mediating the membrane detachment of bacterial lipoproteins depending on their sorting signals.. FEBS Lett.

[30] Yakushi T, Masuda K, Narita S, Matsuyama S, Tokuda H (2000). A new ABC transporter mediating the detachment of lipid-modified proteins from membranes.. Nat Cell Biol.

[31] Okuda S, Tokuda H (2009). Model of mouth-to-mouth transfer of bacterial lipoproteins through inner membrane LolC, periplasmic LolA, and outer membrane LolB.. Proc Nath Acad Sci U S A.

[32] Joseph P, Fantino JR, Herbaud ML, Denizot F (2001). Rapid orientated cloning in a shuttle vector allowing modulated gene expression in Bacillus subtilis.. FEMS Microbiol Lett.

[33] Sambrook J, Russell D (2001). *Molecular cloning: A laboratory manual,* 3rd edition..

[34] Guérout-Fleury AM, Shazand K, Frandsen N, Stragier P (1995). Antibiotic-resistance cassettes for B*acillus subtilis*.. Gene.

[35] Bernard R, El Ghachi M, Mengin-Lecreulx D, Chippaux M, Denizot F (2005). BcrC from *Bacillus subtilis* acts as an undecaprenyl pyrophosphate phosphatase in bacitracin resistance.. J Biol Chem.

[36] Altschul SF, Madden TL, Schäffer AA, Zhang J, Zhang Z (1997). Gapped blast and psi-blast: a new generation of protein database search programs.. Nucleic Acids Res.

[37] Edgar RC (2004). Muscle: multiple sequence alignment with high accuracy and high throughput.. Nucleic Acids Res.

[38] Philippe H (1993). Must, a computer package of management utilities for sequences and trees.. Nucleic Acids Res.

[39] Guindon S, Gascuel O (2003). A simple, fast, and accurate algorithm to estimate large phylogenies by maximum likelihood.. Syst Biol.

[40] Ronquist F, Huelsenbeck JP (2003). MrBayes 3: Bayesian phylogenetic inference under mixed models.. Bioinformatics.

