# Supplementary figures and images for: A New Highly Conserved Antibiotic Sensing/Resistance Pathway in Firmicutes Involves an ABC Transporter Interplaying with a Signal Transduction System
Source: PLoS One. 2011 Jan 19;6(1):e15951. doi: 10.1371/journal.pone.0015951 (PMC3023708; doi:10.1371/journal.pone.0015951)

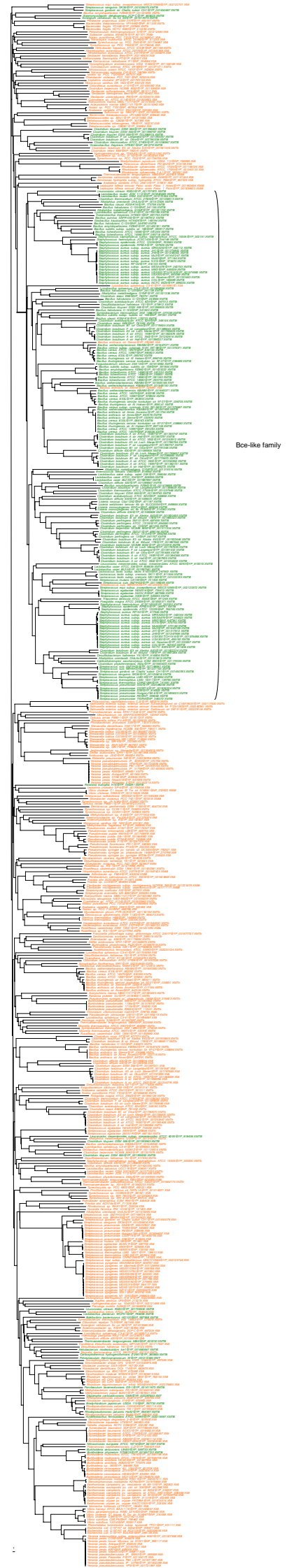

Supplement: Figure S1 — Phylogeny of BceS homologues. Maximum Likelihood tree showing the 579 BceS homologues retrieved from complete genomes. Sequences with the name in green correspond to kinases that harbour exactly 2 TM separated by a short linker (<12 amino acids), whereas sequences with the name in orange correspond to BceS with different characteristics (more or less TM, longer linker, etc). The great majority of these sequences are clustered together, which indicates that this characteristic was present in the ancestor and conserved during the evolution of this group. The few sequences outside the clusters correspond to sporadic convergences occurring during the evolution of this large family. The scale bar gives the average number of substitutions per site. (PDF) [file pone.0015951.s001.pdf]

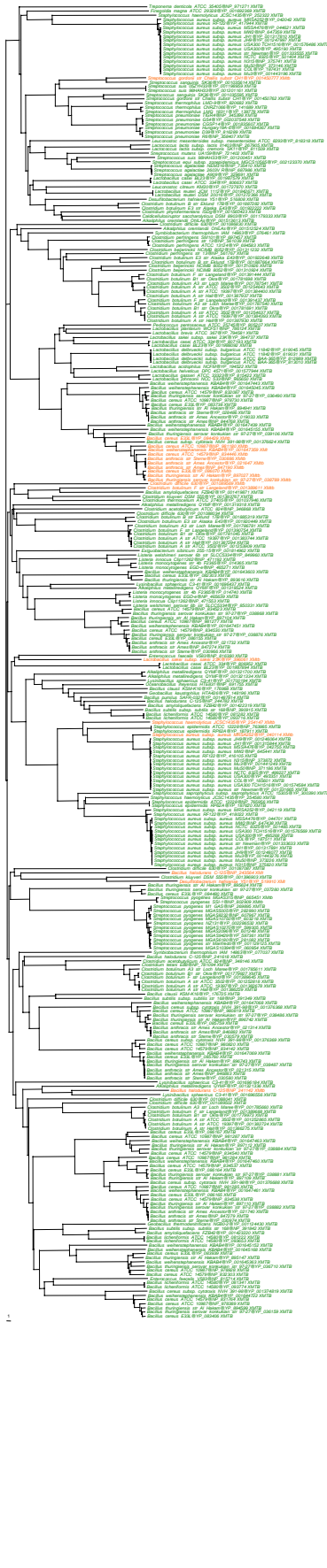

Supplement: Figure S2 — Phylogeny of BceB homologues. Maximum likelihood tree showing the 314 BceB homologues retrieved from complete genomes. Sequences with the name in green correspond to MSD that harbour exactly 10 TM, and TM7 and TM8 are separated by a long extracytoplasmic loop (>197 amino acids), whereas sequences with the name in orange correspond to BceB with different characteristics (more or less TM, longer linker, etc). The scale bar gives the average number of substitutions per site. (PDF) [file pone.0015951.s002.pdf]

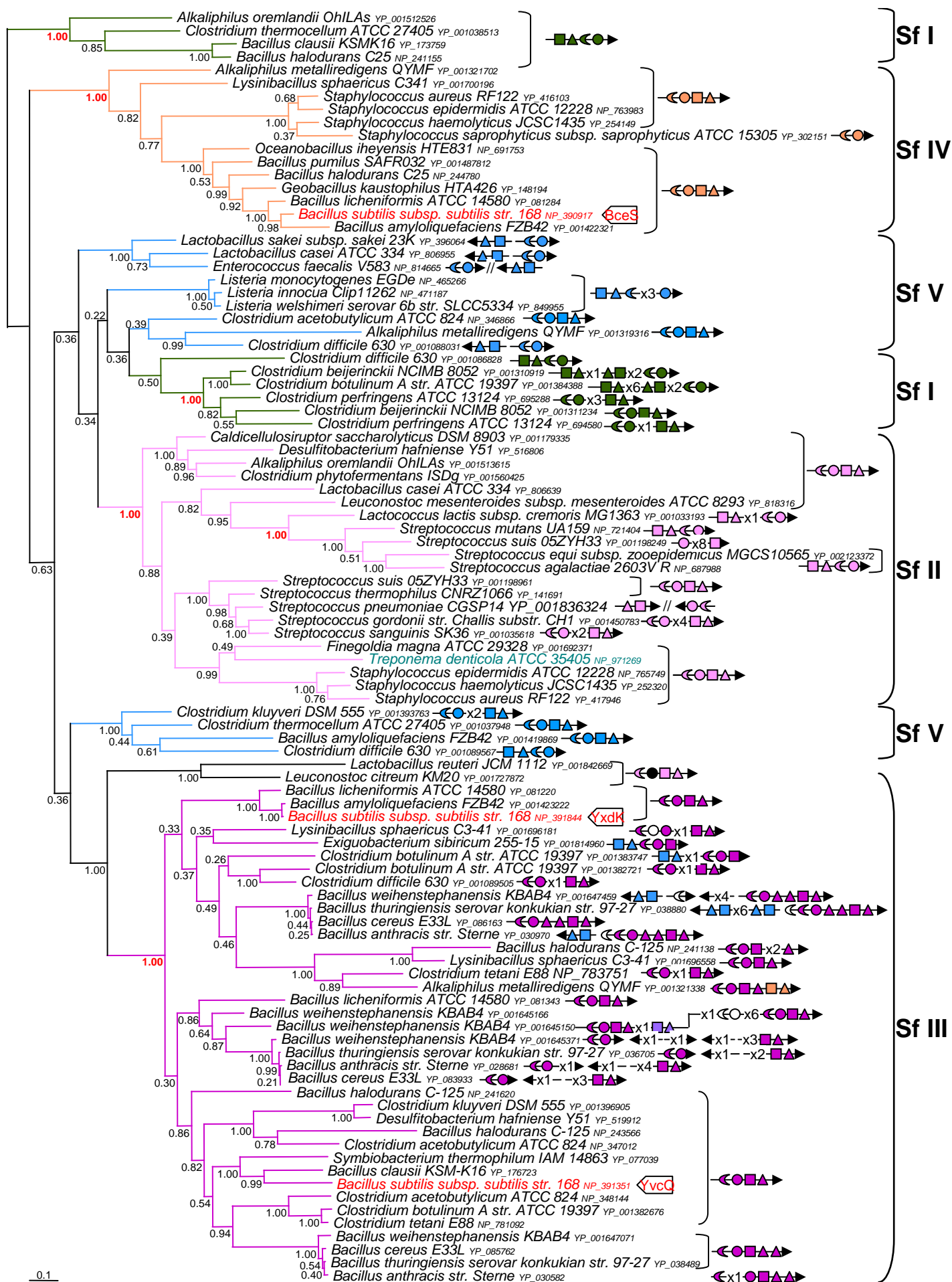

Supplement: Figure S3 — Phylogeny of the BceS-like proteins (kinase components). Bayesian tree showing the relationships in a subsample of 98 BceS sequences. The accession number of each sequence is provided. Numbers at nodes are posterior probabilities. The scale bar gives the average number of substitutions per site. For details about colours and symbols, see the legend to Figure 5. The length of the alignment used to construct the tree was 171 residues. (PDF) [file pone.0015951.s003.pdf]

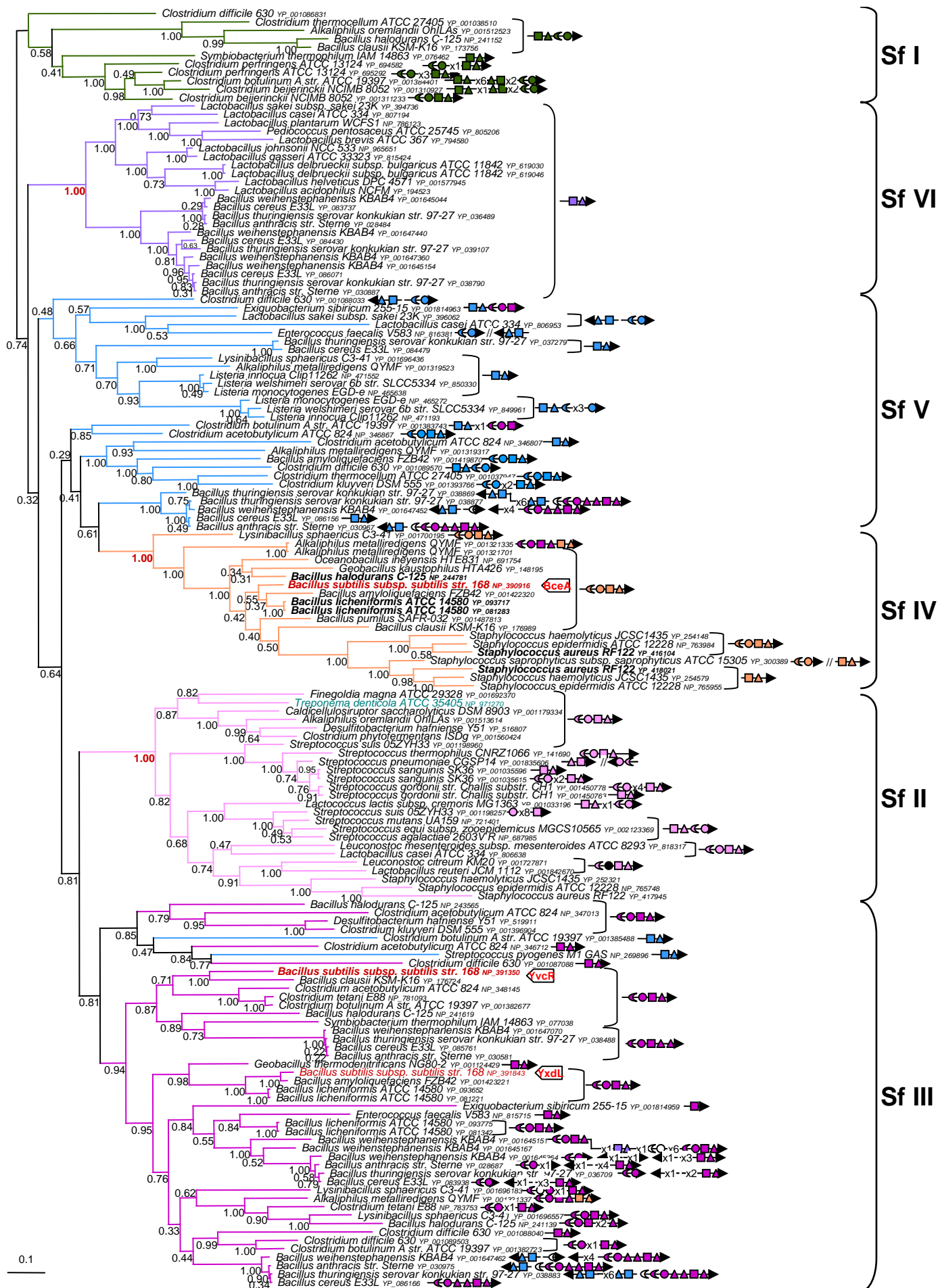

Supplement: Figure S4 — Phylogeny of the BceA-like proteins (NBD components). Bayesian tree showing the relationships in a subsample of 152 BceA sequences. The accession number of each sequence is provided. Numbers at nodes are posterior probabilities. The scale bar gives the average number of substitutions per site. For details about colours and symbols, see the legend to Figure 5. The length of the alignment used to construct the tree was 205 residues. BceA-like proteins used for recombinant strain construction were indicated in bold characters. (PDF) [file pone.0015951.s004.pdf]

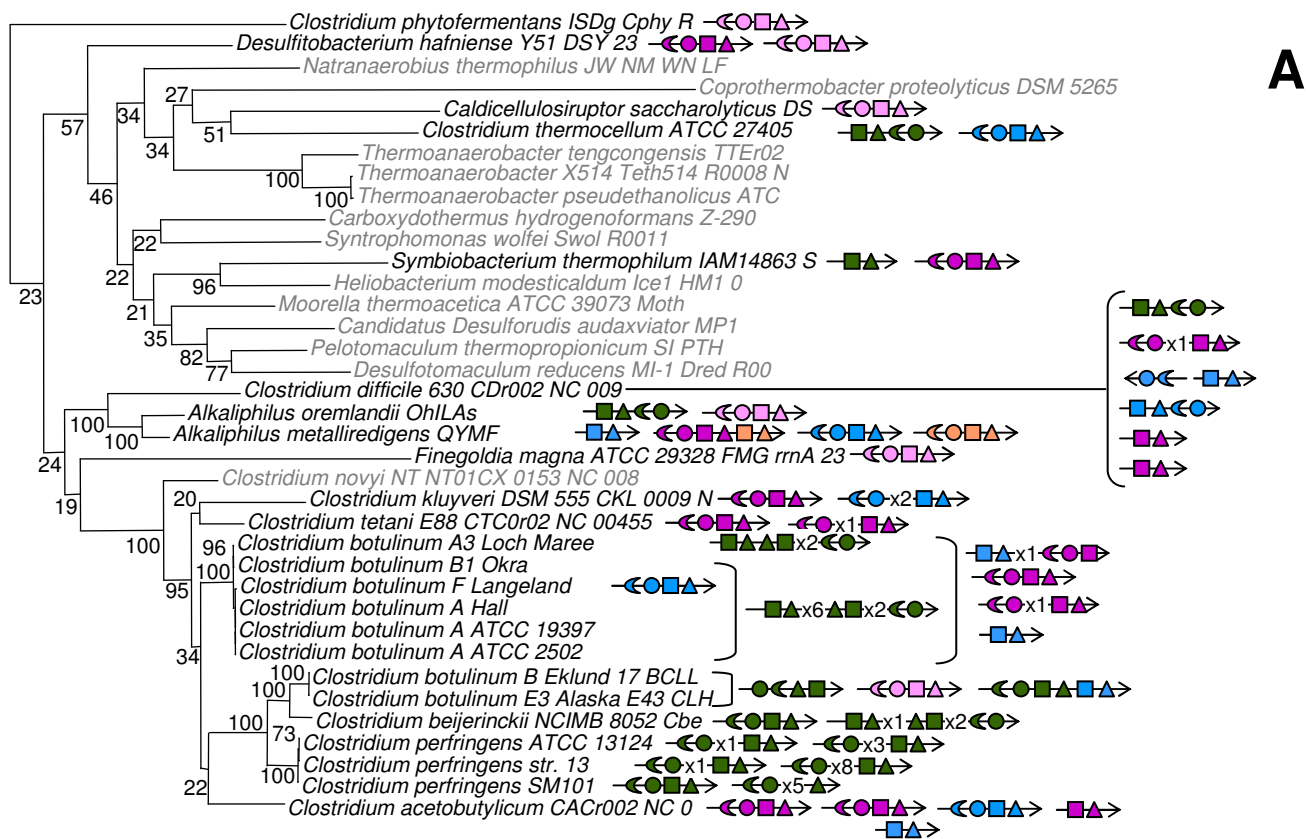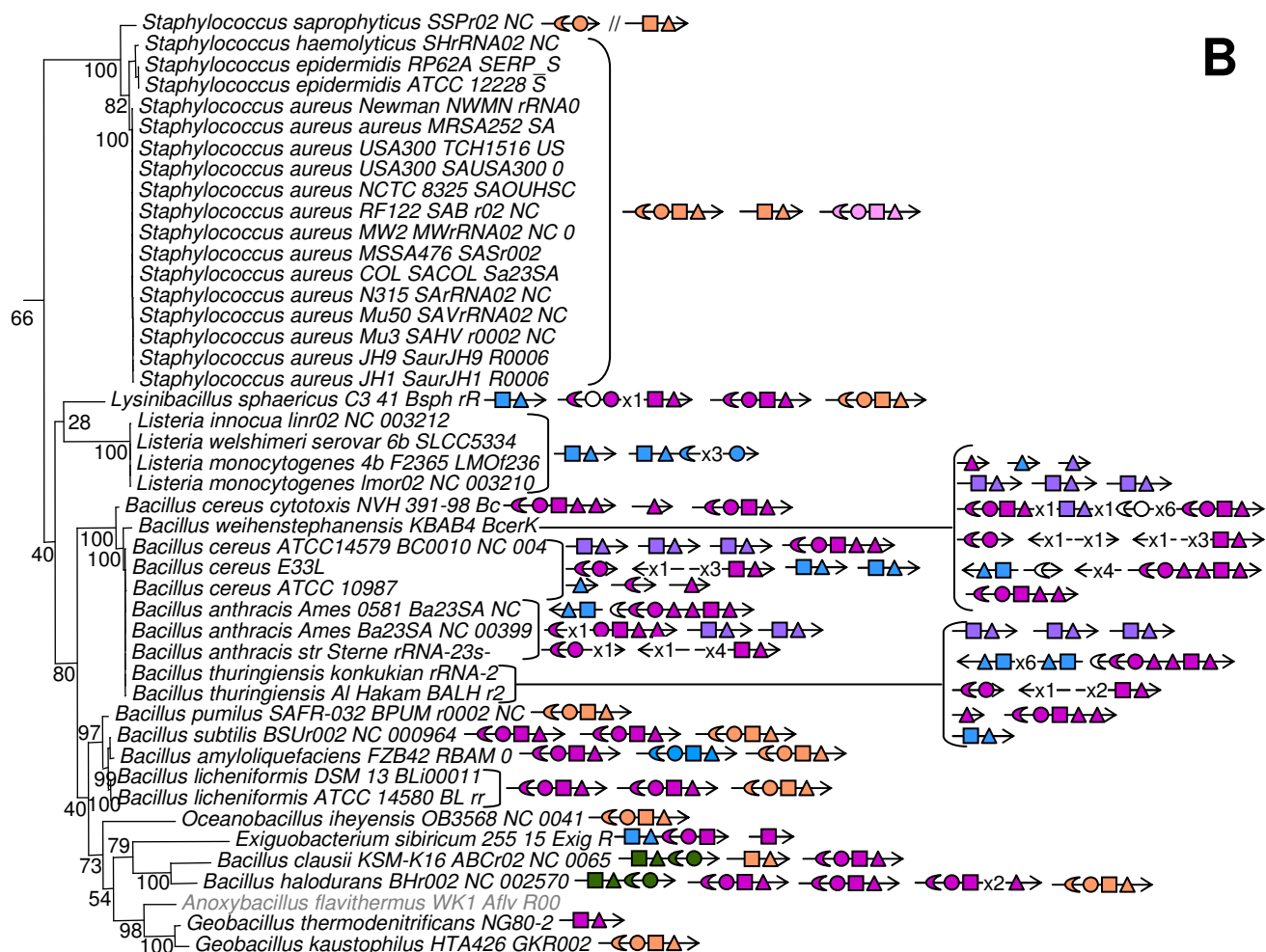

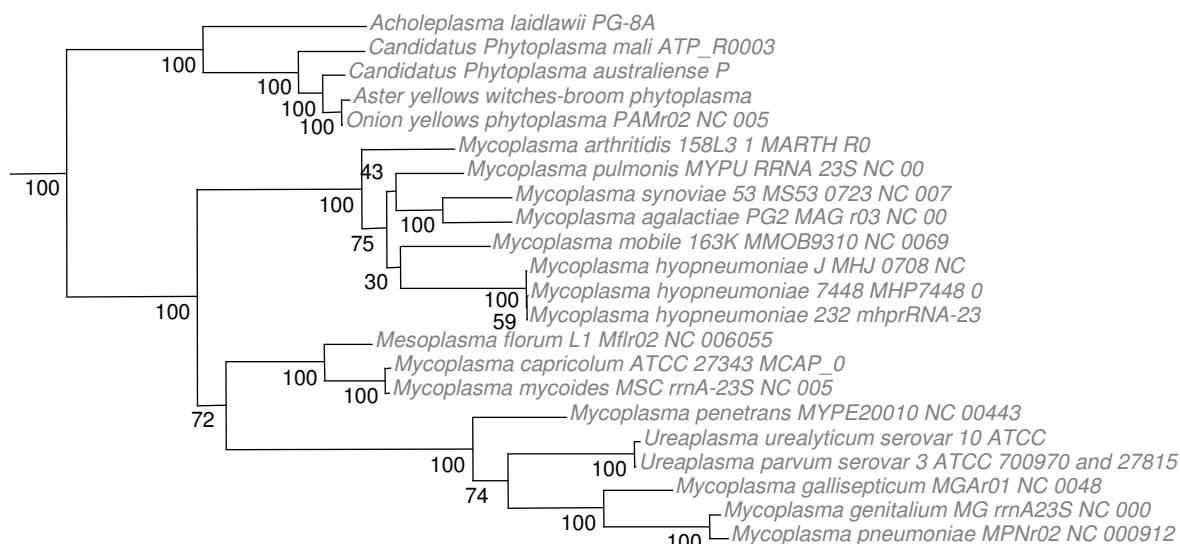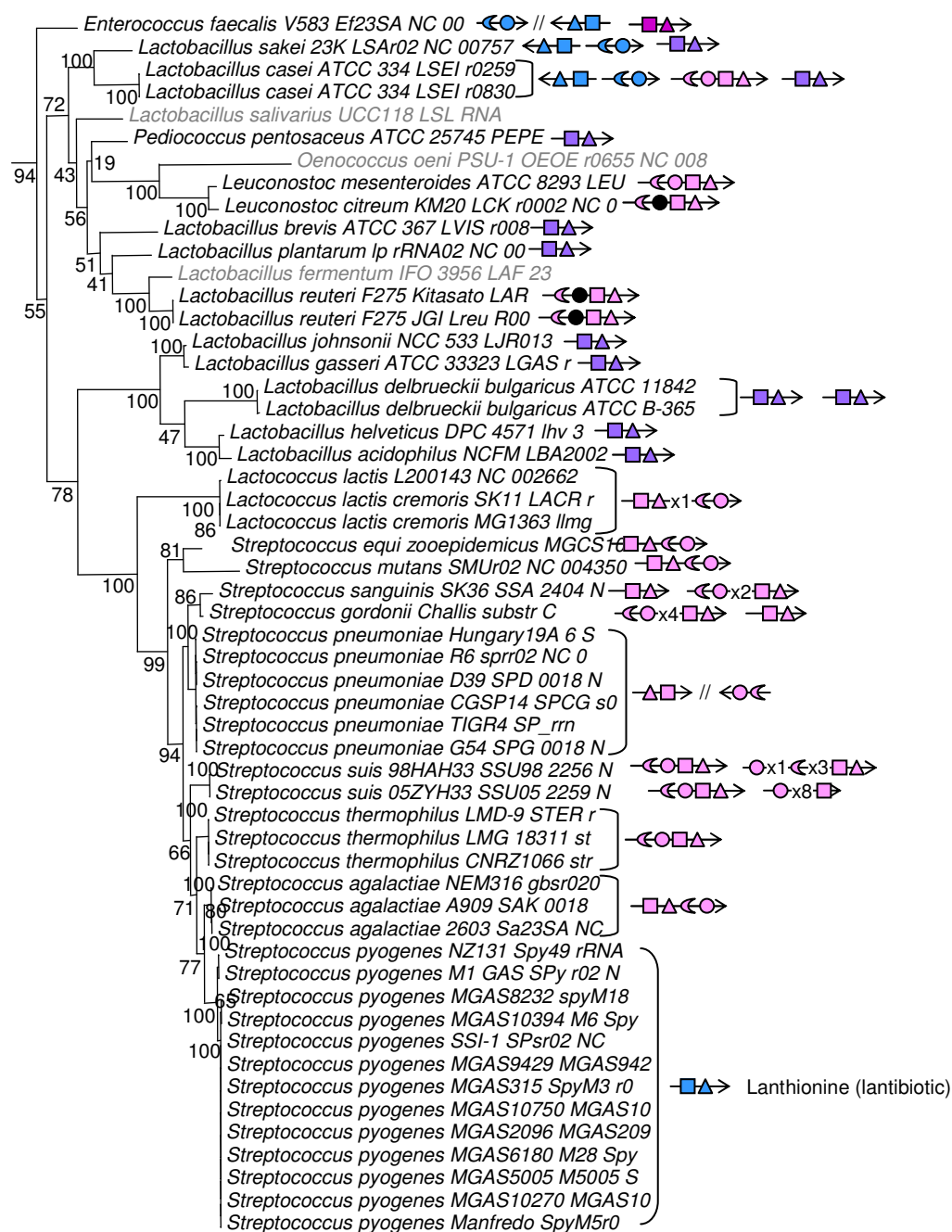

Supplement: Figure S5 — Bce-like repertories. Type and distribution of Bce-like systems in the four main Firmicutes lineages: (A) Clostridiales, (B) Bacillales, (C) Mollicutes and (D) Lactobacillales. For details about symbols and colours, see the legend of Figure 5. (TIF) [file pone.0015951.s005.tif]

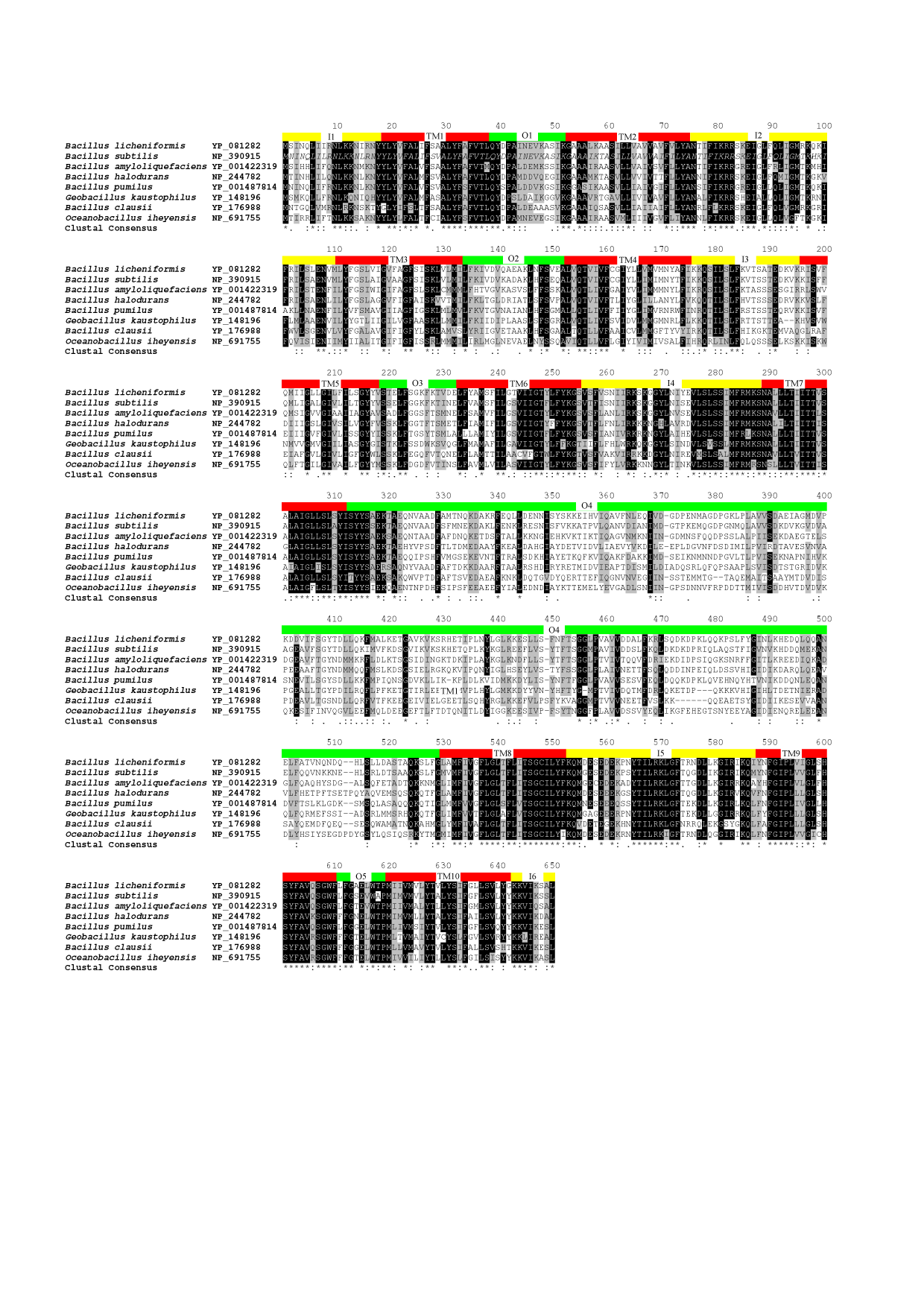

Supplement: Figure S6 — Alignment of the B. subtilis BceB sequence with its seven closest relatives belonging to subfamily IV. Regions designated by I1 to I6 are intracellular segments, those annotated O1 to O5 correspond to extracellular loops (O4 is the large loop), and TM stands for transmembrane segments. In the line entitled “Clustal Consensus”, stars correspond to positions harbouring residues that are different but that have similar features. In this alignment, the region corresponding to O4 is the least highly conserved. (TIF) [file pone.0015951.s006.tif]

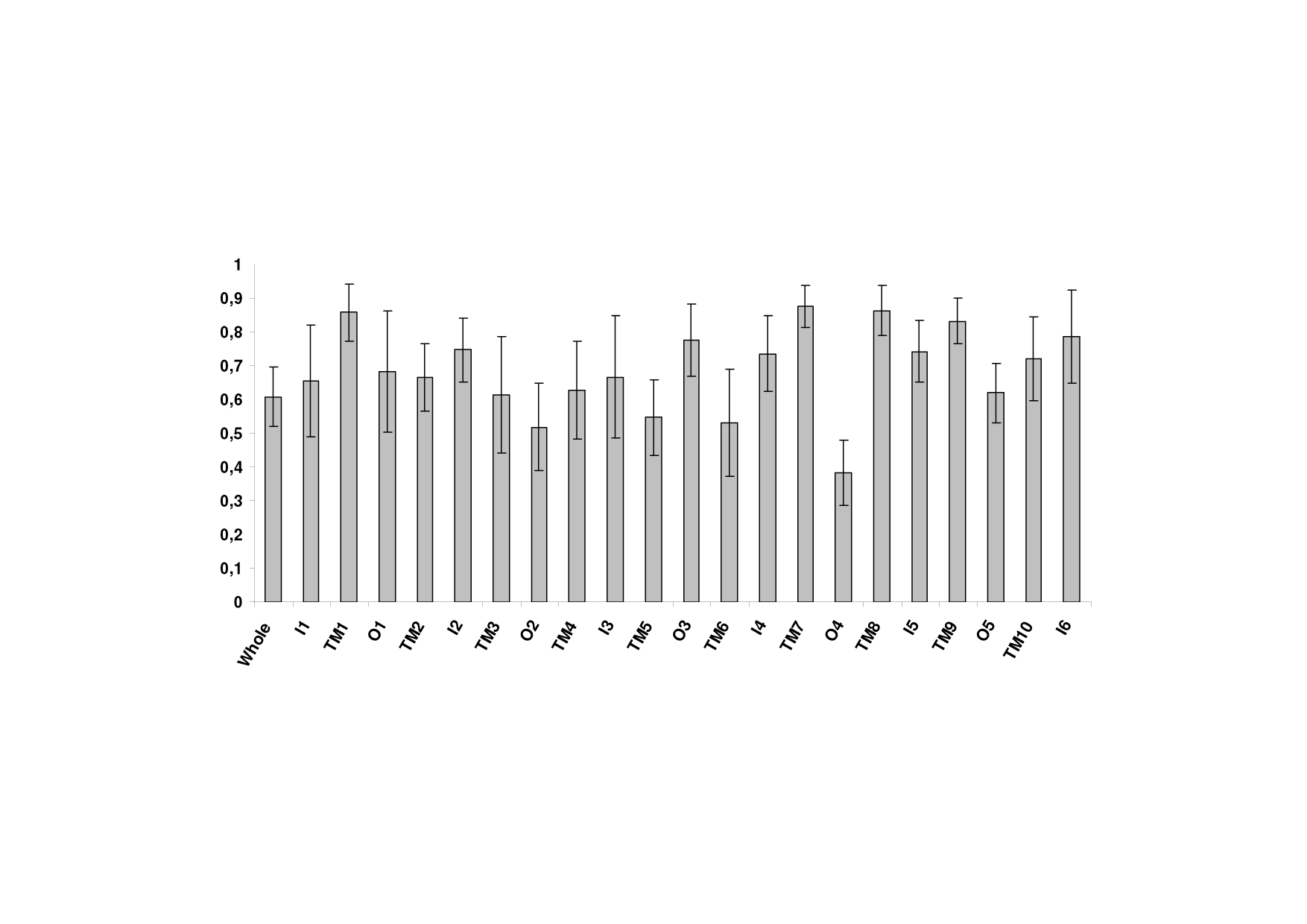

Supplement: Figure S7 — Similarities between B. subtilis BceB regions and those of the closest BceB-like homologs. The Y axis gives the evolutionary distances and the X axis gives the domain of interest in the BceB-like proteins. Whole: entire proteins, I: intracytoplasmic domains, TM: transmembrane segments, O: extracytoplasmic or outside domains. SD Numbering of the domains corresponds to the predicted topology of BceB-like proteins (Figure S6). In each region of the proteins, the mean distance between the B. subtilis BceB transporter and the seven most closely related BceB-like proteins belonging to subfamily IV (proteins from B. licheniformis, B. amyloliquefaciens, B. halodurans, B. pumilus, G. kaustophilus, B. clausii and O. iheyensis) is indicated. Error bars correspond to standard deviations. (TIF) [file pone.0015951.s007.tif]
