# Supplementary material for: A New Highly Conserved Antibiotic Sensing/Resistance Pathway in Firmicutes Involves an ABC Transporter Interplaying with a Signal Transduction System
Source: PLoS One. 2011 Jan 19;6(1):e15951. doi: 10.1371/journal.pone.0015951 (PMC3023708; doi:10.1371/journal.pone.0015951)
Supplement: Table S2 — Bacterial strains used in this study. (PDF) [file pone.0015951.s009.pdf]

Strains	Genotype	Antibiotic resistance	Origin
Escherischia coli DH5α	F- 80*lacZ M15* (*lacZYA–argF)U169*<br>*deoR recA1endA1 hsdR17 phoA* supE44<br>*-thi-1 gyrA96 relA1*		In vitrogen
B. licheniformis DSM 13 (ATCC 14580)			DSMZ
B. halodurans DSM 18197 (ATCC BAA-125)			DSMZ
Bacillus subtilis 168	*trpC2*		Anagnostopoulos and Spizizen 1961
BSmrs 111 (**168, pDG*bceR*)**	168, pDG*bceR*	Km^r	Joseph et al. 2002
**BSGY005**	168, *amyE::*P*bceA::lacZ*	Cm^r	Ohki et al. 2003
BSmrs 203 (**pDG*bceR*)**	BSGY005, pDG*bceR*	Cm^r, Km^r	This work
BSmrs 293 (**Δ*bceAB***)	BSGY005, P*bceA::spc::yttA*	Spc^r, Cm^r	This work
BSmrs 297 (**Δ*bceAB, pDGbceR***)	293, pDG148*bceR*	Spc^r, Cm^r, Km^r	This work
BSmrs 294 (***bceABBs***$_{ri}$)	BSGY005, P*bceA::his6-bceABBs*::*yttA*	Tc^r, Cm^r	This work
BSmrs 298 (***bceABBs***$_{ri,}$**, pDG*bceR***)	294, pDG*bceR*	Tc^r, Cm^r, Km^r	This work
BSmrs 313 (***ytsCDBl***$_{ri}$)	BSGY005, P*bceA::his6-ytsCDBl::yttA*	Tc^r, Cm^r	This work
BSmrs 318 (***ytsCDBl***$_{ri}$**, pDG*bceR***)	313, pDG*bceR*	Tc^r, Cm^r, Km^r	This work
BSmrs 312 (***bceABBh***$_{ri}$)	BSGY005, P*bceA::his6-bceABBh::yttA*	Tc^r, Cm^r	This work
BSmrs 317 (***bceABBh***$_{ri}$ **, pDG*bceR***)	312, pDG*bceR*	Tc^r, Cm^r, Km^r	This work
Bsmrs 357 (***vraDESa***$_{ri}$)	BSGY005, P*bceA::his6-vraDESa::yttA*	Tc^r, Cm^r	This work
Bsmrs 361 (***vraDESa***$_{ri}$**, pDG*bceR*)**	357, pDG*bceR*	Tc^r, Cm^r, Km^r	This work
Bsmrs 358 (***vraFGSa***$_{ri}$)	BSGY005, P*ceA::his6-vraFGSa::yttA*	Tc^r, Cm^r	This work
Bsmrs 362 (***vraFGSa***$_{ri}$**, pDG*bceR*)**	358, pDG*bceR*	Tc^r, Cm^r, Km^r	This work
BSmrs 295 (***bceBBs*Δloop**)	BSGY005, P*bceA::his6-bceABBs*Δloop*::yttA*	Tc^r, Cm^r	This work

BSmrs 299 (**_bceBBs_Δloop, pDG_bceR_**)	295, pDG*bceR*	Tc^r, Cm^r, Km^r	This work
BSmrs 336 (**_ytsDBl_Δloop**)	BSGY005, P*bceA::his6-ytsCDBl*Δ*loop::yttA*		
BSmrs 337 (**_ytsDBl_Δloop, pDG_bceR_**)	336, pDG*bceR*		
BSmrs 296 (**_bceB_loopBceB_Bs_**$_{ri}$)	BSGY005, P*bceA::his6-bceABBs*loopBceB*Bs*$_{ri}$::*yttA*	Tc^r, Cm^r	This work
BSmrs 300 (**_bceB_loopBceB_Bs_**$_{ri}$**, pDG_bceR_**)	296, pDG*bceR*	Tc^r, Cm^r, Km^r	This work
BSmrs 315 (**_bceBBs_loopYtsD_Bl_**$_{ri}$)	BSGY005, P*bceA::his6-bceABBs* loopYtsD*Bl*$_{ri}$::*yttA*	Tc^r, Cm^r	This work
BSmrs 320 (**_bceBBs_loopYtsD_Bl_**$_{ri}$**, pDG_bce_**)	315, pDG*bceR*	Tc^r, Cm^r, Km^r	This work
BSmrs 314 (**_bceBBs_loopBceB_Bh_**$_{ri}$)	BSGY005, P*bceA::his6-bceABBs* loopBceB*Bh*$_{ri}$::*yttA*	Tc^r, Cm^r	This work
BSmrs 319 (**_bceBBs_loopBceB_Bh_**$_{ri}$**,pDG_bceR_**)	314, pDG*bceR*	Tc^r, Cm^r, Km^r	This work
Bsmrs 316 (**_bceBBs_loopYvcS_Bs_**$_{ri}$)	BSGY005, P*bceA::his6-bceABBs* loop YvcS*Bs*$_{ri}$::*yttA*	Tc^r, Cm^r	This work
Bsmrs 321 (**_bceBBs_loopYvcS_Bs_**$_{ri}$**,pDG_bceR_**)	316, pDG*bceR*	Tc^r, Cm^r, Km^r	This work

To facilitate understanding strain names, indicated in bold characters, are used throughout the text and tables.

Anagnostopoulos C, Spizizen J. 1961. Requirements for transformation in *Bacillus subtilis*. J Bacteriol **81**: 741-746.
Joseph P, Fichant G, Quentin Y, Denizot F. 2002. Regulatory relationship of two-component and ABC transport systems and clustering of their genes in the *bacillus/clostridium* group, suggest a functional link between them. J Mol Microbiol Biotechnol **4**: 503-513.
Ohki R, Giyanto, Tateno K, Masuyama W, Moriya S, Kobayashi K, Ogasawara N. 2003. The BceRS two-component regulatory system induces expression of the bacitracin transporter, BceAB, in *Bacillus subtilis*. Mol Microbiol **49**: 1135-1144.
