# Supplementary material for: A New Highly Conserved Antibiotic Sensing/Resistance Pathway in Firmicutes Involves an ABC Transporter Interplaying with a Signal Transduction System
Source: PLoS One. 2011 Jan 19;6(1):e15951. doi: 10.1371/journal.pone.0015951 (PMC3023708; doi:10.1371/journal.pone.0015951)
Supplement: Table S3 — Plasmids used in this study. (PDF) [file pone.0015951.s010.pdf]

Plasmids	Features, resistance	Références
pDG*bceR*	IPTG inducible *bceR* expression, Ap^r, Km^r	Joseph et al. 2002
pbSy	Ap^r, Spc^r	This work
pbTy	Ap^r, Tc^r	This work
pbTy *bceAB*	Ap^r, Tc^r	This work
pbTy *ytsCDBl*	Ap^r, Tc^r	This work
pbTy *bceABBh*	Ap^r, Tc^r	This work
pbTy vraDE*Sa*	Ap^r, Tc^r	This work
pbTy vraFG*Sa*	Ap^r, Tc^r	This work
pbTy *bceABBs*Δloop	Ap^r, Tc^r	This work
pbTy *ytsCDBl*Δloop	Ap^r, Tc^r	This work
pbTy *bceA,bceB*loopBceB*Bs*	Ap^r, Tc^r	This work
pbTy *bceA, bceB*loopYtsD*Bl*	Ap^r, Tc^r	This work
pbTy *bceA, bceB*loopBceB*Bh*	Ap^r, Tc^r	This work
pbTy *bceA, bceB*loopYvcS*Bs*	Ap^r, Tc^r	This work

Joseph P, Fichant G, Quentin Y, Denizot F. 2002. Regulatory relationship of two-component and ABC transport systems and clustering of their genes in the *bacillus/clostridium* group, suggest a functional link between them. J Mol Microbiol Biotechnol **4**: 503-513.
