# Supplementary material for: A New Highly Conserved Antibiotic Sensing/Resistance Pathway in Firmicutes Involves an ABC Transporter Interplaying with a Signal Transduction System
Source: PLoS One. 2011 Jan 19;6(1):e15951. doi: 10.1371/journal.pone.0015951 (PMC3023708; doi:10.1371/journal.pone.0015951)
Supplement: Table S4 — List of oligonucleotides used in this study. (PDF) [file pone.0015951.s011.pdf]

Name	Sequence
Pbcea1	5'-GGGCATTGATCCAAAAGATGTACC-3'
Pbcea2-pml	5'-CGCCC ACGTGATGATGATGATGGTGCATTTACGCAG TCTCCTTTAATTG-3'
Tet1-asc	5'-TCATCATCATCACGTGGGCGCGCCAATGGTGCAGGT TGTTCTCAATG-3'
Tet2-lin	5'-AGGATCAATTTTGAACTCTCTCCC-3'
yttA1-tet	5'-GGGAGAGAGTTCAAAATTGATCCTGAAGGTCATT AAATCGTCGTTGTG-3'
ytta2	5'-AGAAGATGCAAAAACGGCAAAGG-3'
spec-asc	5'-TGGCGCGCCGATTTTCGTTCGTGAATACATG-3'
spec-bst	5'-ACAGGTTACCACCAATTAGAATGAATATTTCCC-3'
bceABBs-pml	5'-GTGATTTTAGAAGCGAATAAAATTCG-3'
bceABBs-asc	5'-TTGGCGCGCCAGGAAGCAGAAACGGCATCTACC-3'
ytsCDBl-pml	5'-ATGATTTTAGAAGCGAAAAAAATACG-3'
ytsCDBl-pml	5'-TTGGCGCGCCGTCCGATGGGCTTTTGATTATGC-3'
bceABBh-pml	5'-GCAATACTTGAAGCGACAAACATTC-3'
bceABBh-asc	5'-TTGGCGCGCCTTTAAGTGAAGAGCAGCGGCAAC-3'
loop1/FseBbv	5'-GCTGAGGACAATGAGGCCGGCCGAGTAATACGA GATGTAAGCAAG-3'
loop2/FseBbv	5'-GCCGGCCTCATTGTCCTCAGCTCACTGTTTGGTAT GGTGATGTTC-3'
loop-BceBBs_fse	5'-TCGGCCGGCCTTTCGGAAAAGACCGCTGAACAAAAT G-3'
loop-BceBBs_bbv	5'-GAGCTGAGGACTTTTTGCGCAGCGCTTGTATCTAG-3'
loop-ytsD-Bl_fse	5'-TCGGCCGGCCTTGCAGAGAAAACCGCCGAACAAAAC-3'
loop-ytsD-Bl_bbv	5'-GAGCTGAGGACTTTTTGAGCTGTGCTTGCATCAAG-3'
loop-BceBBh_fse	5'-TCGGCCGGCCTTGCGGAAAAAACAGCGGAACATTATG-3'
loop-BceBBh_bbv	5'-GAGCTGAGGACCTTCTGACTTTGGCTCATTTCAAC-3'
vraFG_PmlI	5’-CACAAGAAGTGTTGCGAGATATC-3’
vraFG_AscI	5’-TTGGCGCGCCGCTTCCAGTATAGTTCGTGAATCC-3’
vraDE_PmlI	5’-ACGATATTATCAGTGCAACATGTTTC-3’
vraDE_AscI	5’-TTGGCGCGCCGCAATGACGTTATACCAAGTTACAGTC-3’
